# Supplementary material for: Resurrecting ancestral antibiotics: unveiling the origins of modern lipid II targeting glycopeptides
Source: Nat Commun. 2023 Nov 29;14:7842. doi: 10.1038/s41467-023-43451-4 (PMC10687080; doi:10.1038/s41467-023-43451-4)
Supplement: Supplementary file 1 — Supplementary Information [file 41467_2023_43451_MOESM1_ESM.pdf]

Resurrecting Ancestral Antibiotics: Unveiling the Origins of Modern Lipid II Targeting Glycopeptides

Hansen, Adamek & Iftime et al.

Supplementary Information

Supplementary Tables ………………………………………………… **Page 2**
Supplementary Figures ………………………………………………… **Page 12**
Supporting Note 1 ……………………………………………………….. **Page 61**
Supplementary References ...……………………………………………… **Page 63**

Supplementary Tables

Table S1. Sequences from all glycopeptide clusters used in this work

Bacterial strain (cluster associated with known structure)	Glycopeptide	Accession number [contigID]	Position
**Actinoplanes teichomyceticus*	Teicoplanin	AJ605139.1	1-89,713
**Actinoplanes* sp. *ATCC 53533 UK-68,597*	UK-68,597	KF192710.1	1-80,908
**Amycolatopsis balhimycina DSM 44591*	Balhimycin	ARBH00000000.1	11,773 - 77,557
** Amycolatopsis coloradensis DSM 44225*	Avoparcin	NZ_MQUQ00000000.1	16,064 - 85,438
**Amycolatopsis decaplanina DSM 44594*	Decaplanin	AOHO00000000.1	16,059 - 82,438
**Amycolatopsis japonica MG417-CF17, DSM 44213*	Ristocetin	CP008953.1	6,817,866-6,885,328
**Amycolatopsis keratiniphila* ssp. *nogabecina DSM 44586*	Actinoidin	MQUP00000000.1 [MQUP01000022.1]	96,719 - 165,358
**Amycolatopsis lurida NRRL 2430*	Ristocetin	JFBM00000000.1 [JFBM01000018.1]	18,952 - 88,068
**Amycolatopsis orientalis A82846*	Chloroeremomycin	AL078635.1<br><br>AJ223999.1<br>AJ223998.1	29,715-42,369<br>1-34,244<br>1-26,657
**Amycolatopsis orientalis B-37*	Norvancomycin	CP016174.1	7,726,012 - 7,793,138
**Amycolatopsis orientalis DSM 40040*	Vancomycin	NZ_ASJB00000000.1 [ASJB01000062.1]	74,177 - 142,304
**Amycolatopsis keratiniphila HCCB10007*	Vancomycin	CP003410.1	1,533,274 - 1,598,414
**Amycolatopsis* sp. *MJM2582*	Ristocetin	NZ_JPLW00000000.1 [JPLW01000007.1]	284,850 - 353,956
**Nonomuraea* sp. *ATCC 39727*	A40926	LT559118.1	2,231,845 - 2,302,698
**Nonomuraea coxensis DSM 45129*	A50926	CP068985.1	2,889,258 - 2,825,582
Nonomuraea sp. *ATCC 55076*	Kistamicin	CP017717.1	11,980,264 - 12,028,278
Streptomyces lavendulae	Complestatin	AF386507.1	1-55,927
**Streptomyces* sp. *WAC1420*	Pekiskomycin	JX026280.1	1-85,659
**Streptomyces* sp. *WAC4229*	Pekiskomycin	KC688274.1	1-69,000
**Streptomyces toyocaensis NRRL 15009*	A47934	JFCB00000000.1	254,209 - 318,618

Bacterial strain (predicted heptapeptide GPA cluster)	Accession number [contigID]	Position
Actinokineospora auranticolor YU 961-1	PTIX00000000.1 [PTIX01000004.1]	41,464 - 112,059
**Actinoplanes rectilineatus NRRL B-16090*	NZ_JZKF00000000.1 [JZKF01000002.1]	148,291 - 227,933
**Actinoplanes subtropicus NRRL B-24665*	NZ_JOJL00000000.1	74,259 - 173,942
**Amycolatopsis alba DSM 44262*	ARAF00000000.1	3,130,701- 3,196,280
**Amycolatopsis azurea DSM 43854*	MUXN00000000.1 [MUXN01000025.1]	140,113 - 214,581
**Amycolatopsis regifaucium DSM 45072*	LOBU00000000.2 [LOBU02000007.1]	258,746 - 327,583
**Amycolatopsis* sp. *BJA-103*	CP017780.1	9,256,687 - 9,327,913
**Amycolatopsis* sp. CB00013	LIWC00000000.1 [LIWC01000005.1]	370,163 - 440,369
**Amycolatopsis* sp. *H5*	NZ_NMUL00000000.1 [NMUL01000010.1]	3,809 - 64,939
**Amycolatopsis* sp. *WAC1375*	JX576190.1<br>NZ_QHHW00000000.1<br>[QHHW01000063.1]<br>[QHHW01000028.1]	1,197 -65,229<br><br>1 - 14,290<br>47,073 - 96,968
**Amycolatopsis thailandensis JCM 16380*	NZ_NMQT00000000.1<br>[NMQT01000045.1]<br>[NMQT01000068.1]	 2,812 - 45,094<br>35,102 - 44,799
**Kibdelosporangium aridum DSM 43828*	FWXV00000000.1 [FWXV01000011.1]	185,629 - 248,622
Kitasatospora aureofaciens NRRL B-2658	LGUY00000000.1 [LGUY01000259.1]	96,539 - 152,514
Nocardia terpenica NC_YFY_NT001	CP023778.1	6,951,984 - 7,011,554
Streptomyces albus ssp. *albus NRRL F-4371 P384*	LMZE00000000.1 [LMZE01000068.1]	80,881 - 133,546
Streptomyces avermitilis MA-4680	BAVY01000000.1 [BAVY01000022.1]	74,634 - 127,245
Streptomyces fradiae ATCC 19609	NAD01000004.1	32,310 - 115,226
Streptomyces rimosus ssp. *rimosus NRRL WC-3869 P248*	LGCQ00000000.1 [LGCQ01000230.1]	80,544 - 134,996
Streptomyces sp. *CB02923*	LWKZ00000000.1 [LWKZ01000024.1]	232,283 - 288,381
Streptomyces sp. *KS_5*	FNTE00000000.1 [FNTE01000002]	507,624 - 554,104
Streptomyces sp. *PAN_FS17*	FNTN00000000.1 [FNTN01000001.1]	9,885,228 - 9,937,558
Streptomyces varsoviensis NRRL ISP-5346	JOBF00000000.1 [JOBF01000003]	182,299 - 222,445
Streptomyces xinghaiensis S187	CP023202.1	513,715 - 567,464
Uncultured bacterium esnapd15	KF264554.1	15,287 - 78,611
Uncultured bacterium esnapd26	KF264565.1	14,565 - 73,288
Uncultured organism CA37	HM486074.1	6,152 - 101,100
Uncultured organism CA878	HM486075.1	26,679 - 103,201
Uncultured organism CA915	HM486076.1	12,871 - 98,753
Uncultured soil bacterium clone B128 VEG	EU874252.1	1,001 - 73,083
Uncultured soil bacterium clone D30 TEG	EU874253.1	101 - 51,647

Bacterial strain (predicted noncanonical GPA cluster)	Accession number [ContigID]	Position
Actinobacteria bacterium OK074	LJCV00000000.1 [LJCV01000261.1]	24 - 53,709
Streptomyces sp. *CNY243 C594*	ARHU00000000.1 [NZ_KB897732.1]	9,557 - 65,280

* Sequences used for ancestral sequence reconstruction

Table S2. Bacterial strains and plasmids used in this work

Strains	Characteristics	Reference
E. coli NovaBlue	*recA*1, *endA*1, *gyrA*96, *thi*-1, *hsdR*17 (rK12$^-$,mK12$^+$) *supE*44, *relA*1, *lac* [F', *pro*AB, *lacI*^{q}, *lacZ*ΔM15, Tn*10*] (Tet^R)	Novagen
E. coli HST08 <br>*(Stellar competent cells)*	*F-, endA1, supE44, thi-1, recA1, relA1, gyrA96, phoA, Φ80d lacZΔ M15, Δ(lacZYA-argF) U169, Δ(mrr-hsdRMS-mcrBC), ΔmcrA, λ-*	Takara Bio
E. coli ET12567	DNA methylation deficient donor strain for conjugation: F2*dam*13::Tn*9, dcm*-6, *hsdM, hsdR, recF143, zjj-202::*Tn*10, galK2, galT22 ara-14, lacY1,xyl-5, leuB6, thi-1, tonA31, rpsL136, hisG4, tsx-78, mtl-1, glnV44*	MacNeil, *et al*., 1992[1]
E. coli BL21 (DE3)	DE3 lysogen contains T7 polymerase upon IPTG induction. This strain is eficient of lon and omp-t proteases and is therefore suitable for expression of non-toxic genes.	Novagen
**Plasmids**		
pJet1.2/blunt	rep(pMB1), bla(AmpR), eco47IR, PlacUV5, T7 RNA polymerase promoter. Part of the CloneJET™ PCR Cloning Kit used for high efficiency cloning of PCR products	Thermo Fisher Scientific Inc.
pUB307	self-transmissible plasmid that mobilizes other plasmids *in trans* for DNA transfer into hosts: RP4, kan^R	Bennett, et al., 1977[2]
6pGUSA21	Promoter probe vector, pSETGUS with deleted KpnI fragment containing *tipA* promoter. *gusA*, Δ*int*, Δ*attB*, MCS from pUC21. Used for gene inactivation	Myronovskyi et al., 2011[3]
pGUSA21_RistoKO	pGUSA21 deletion plasmid of *nrps* genes containing 1.3/1.5 kb flanking regions up-and downstream of the *rpsD-rpsA* genes	This study
p3SV	Cloning vector	Lab stock
pDM	p3SV containing the four synthetic *nrps* genes	ATG:synthetics (Merzhausen, Germany
pDI1	p3SV containing the four synthetic *nrps* genes and an additional SP44* promoter	This study
pBHH_bHTyr_KO	pGUSA21 deletion plasmid of the *oxyD, rpsE* and *bhp* genes containing 1.5 kb flanking regions each up-and	This study

	downstream of the *oxyD,* and *bhp* genes	
pIJ_bbr	*ermE** promoter,[4] ΦBT1 phage integration site, oriT from RK2, *bbr* regulatory gene, hyg^R	Lab stock (derived from pIJ)[5]
pHIS17	pBR322-derived plasmid encoding a C-terminal 6xHis tag. The vector backbone is 2608 bp and has the antibiotic resistance gene encoding the ampicillin resistance (AmpR) protein	Kunzelmann and Webb, 2009[6]
pHIS17_ Tcp9_$A1_{tei}$	pHIS17 containing $A1_{tei.}$ Residues 9-492 from Tcp9	This study
pHIS17_ Tcp9_$A1_{core\text{-}tei}$	pHIS17 containing $A1_{core\text{-}tei}$. Residues 9-398 from Tcp9	This study
pHIS_ANC4_$A1_{core}$	pHIS17 containing the truncated synthetic ANC4 gene for expression of residues 1-391.	This study
pCDF-1b	pCDF-1b is 3621 bp and designed for co-expression of target genes with pET series vectors. The vector encodes bacterial resistance to spectinomycin/streptomycin.	EMD Millipore
pCDF-1b_Tcp13	pCDF-1b containing Tcp13	This study
pOPIN-S	pET28a derived vector and has been generated by the Oxford Protein Production Facility (OPPF). This vector contains an N-terminal 6xHis-SUMO tag.	Assenberg et al., 2008[7]
pOPSTR	pOPIN-S derived vector with the addition of a C-terminal Strep-tag	Kaniusaite, et al., 2019[8]
pOPSTR_ANC1	pOPSTR containing the synthetic ANC1 gene.	This study
pOPSTR_ANC2	pOPSTR containing the synthetic ANC2 gene.	This study
pOPSTR_ANC3	pOPSTR containing the synthetic ANC3 gene.	This study
pOPSTR_ ANC4	pOPSTR containing the synthetic ANC4 gene.	This study

Table S3: Primers used in this study

Primer Name	Sequence	Amplicon length bp	PCR Program
Risto KO UP fw	CATATGAAGGAAAACCGATGAGCAATCCCTT	1304 bp	1. 98°C, 0:30 min<br>2. 98°C, 0:10 min<br>3. 58°C, 0:30 min<br>4. 72°C, 1:00 min<br>5. 72°C, 2:00 min<br>(Steps 2-4 × 32)
Risto KO UP rv	TCTAGATTCTCGTCGGTCGTGAGGTACCGGTT		
Risto KO DO fw	GCATGCCAGCAGGCGTTCGGGCATCTGCTGA	1512 bp	
Risto KO DO rv	AAGCTTTCATCCTCCGTAGACCACGGTGTCC		
*A. jap*Δ-Cyt$_{P450}$ fw	ATCGTGACGTCTTCCTTGGCGATA	3118 bp	1. 98°C, 0:30 min<br>2. 98°C, 0:10 min<br>3. 60°C, 0:30 min<br>4. 72°C, 2:00 min<br>5. 72°C, 2:00 min<br>(Steps 2-4 × 32)
*A. jap*Δ-ABC-rv	CGATCGCGTTCTTCACCGCA		
SP44_OH_fw	TTTTTACGGTTCCTGGCCTTTTGATCATTAATTGTTCACATTCGAACCGTCTCT	218 bp	1. 98°C, 0:30 min<br>2. 98°C, 0:10 min<br>3. 67°C, 0:30 min<br>4. 72°C, 0:10 min<br>5. 72°C, 2:00 min<br>(Steps 2-4 × 32)
SP44_OH_rv	CCGTCAAGATCGACCGCGTGCAGATTGTCAGCCTACTCCTTACTTAGATTAAACAAAATTATTTGT		
PacI GPC_fv	GAACCTCTCGAGGGTCGGG	754 bp	1. 98°C, 0:30 min<br>2. 98°C, 0:10 min<br>3. 57°C, 0:30 min<br>4. 72°C, 0:20 min<br>5. 72°C, 2:00 min<br>(Steps 2-4 × 32
bla vec_rv	ACCCACTCGTGCACCC		
Aj-32060 fw	GAGCTCGAATTCGAAGCTTaagaagcccccatcctcacca	1536 bp	1. 98°C, 0:30 min<br>2. 98°C, 0:10 min<br>3. 67°C, 0:30 min<br>4. 72°C, 0:45 min<br>5. 72°C, 2:00 min<br>(Steps 2-4 × 32
Aj-32060 rv	CTGAACTAGcagcatgatgctccttgagggaaaaggg		
BHH-fw	atcatgctgCTAGTTCAGGGGTGATGTGTCAGCC	3100 bp	1. 98°C, 0:30 min<br>2. 98°C, 0:10 min<br>3. 67°C, 0:30 min<br>4. 72°C, 1:30 min<br>5. 72°C, 2:00 min<br>(Steps 2-4 × 32
BHH-rv	gggCATATGggCATATGAGTGAGGAGCTCCTCT		
Aj-32040 fw	TCATATGccCATATGcccgacgtccgtcc	1560 bp	1. 98°C, 0:30 min<br>2. 98°C, 0:10 min<br>3. 67°C, 0:30 min<br>4. 72°C, 0:45 min<br>5. 72°C, 2:00 min<br>(Steps 2-4 × 32
Aj-32040 rv	GGCGATATCGGATCCATATGccgtccgcctacc		
Aj-32060-d fw	tgtcctccaggagcaggatgccgttcg	1530 bp	1. 98°C, 0:30 min<br>2. 98°C, 0:10 min<br>3. 57°C, 0:30 min<br>4. 72°C, 0:45 min<br>5. 72°C, 2:00 min<br>(Steps 2-4 × 32
Hal-i rv	CAAGTACGGGCAGCTGCGGGT		
pIJ fw	GATGCAGTTGCACCAGGCTG	1443 bp	1. 98°C, 0:30 min<br>2. 98°C, 0:10 min<br>3. 59°C, 0:30 min<br>4. 72°C, 0:45 min<br>5. 72°C, 2:00 min<br>(Steps 2-4 × 32
pIJ rv	CCGACATCGACCAGGCG		
pOPIN-S_STREP fw	CTCGAGAGTGCTTGGAGTCATCC	5613 bp	1. 98°C, 0:30 min<br>2. 98°C, 0:10 min<br>3. 62°C, 0:30 min<br>4. 72°C, 1:50 min<br>5. 72°C, 5:00 min<br>(Steps 2-4 × 34)
pOPIN-S_STREP rv	ACCACCGATCTGTTCGCg		
ANC1 fw	GAACAGATCGGTGGTGTGCTGGACTTGTTTGCACGCC	1482 bp	1. 98°C, 0:30 min<br>2. 98°C, 0:10 min<br>3. 65°C, 0:30 min<br>4. 72°C, 0:30 min<br>5. 72°C, 2:00 min<br>(Steps 2-4 × 34)
ANC1 rv	CCAAGCACTCTCGAGTGTCGCATTGGCGGCAAAATCCG		
ANC2 fw	GAACAGATCGGTGGTGTTCTGGACCTCTTTGCTCGGCA	1482 bp	1. 98°C, 0:30 min<br>2. 98°C, 0:10 min

			3. 63°C, 0:30 min<br>4. 72°C, 0:30 min<br>5. 72°C, 2:00 min<br>(Steps 2-4 × 34)
ANC2 rv	CCAAGCACTCTCGAGGGTCGCATTTGCCGCAAATTCC		
ANC3 fw	GAACAGATCGGTGGTGTATTGGACCTGTTTGCGCGCC	1479 bp	1. 98°C, 0:30 min<br>2. 98°C, 0:10 min<br>3. 63°C, 0:30 min<br>4. 72°C, 0:30 min<br>5. 72°C, 2:00 min<br>(Steps 2-4 × 34)
ANC3 rv	CCAAGCACTCTCGAGTGTGGCATTCGCCGCAAATTCCG		
ANC4 fw	GAACAGATCGGTGGTGTATTGGAACTCTTTGCGCGTCATGT	1491 bp	1. 98°C, 0:30 min<br>2. 98°C, 0:10 min<br>3. 63°C, 0:30 min<br>4. 72°C, 0:30 min<br>5. 72°C, 2:00 min<br>(Steps 2-4 × 34)
ANC4 rv	CCAAGCACTCTCGAGGGTGGCATTGGCCGCGA		
ANC4$_{core}$ fw	GGAGATATACATATGGTATTGGAACTCTTTGCGCGTCA	1203 bp	1. 98°C, 0:30 min<br>2. 98°C, 0:10 min<br>3. 63°C, 0:30 min<br>4. 72°C, 0:25 min<br>5. 72°C, 2:00 min<br>(Steps 2-4 × 34)
ANC4$_{core}$ rv	atgatgatgggatccCTGGTGGTCAACGCGACC		
pHIS17 fw	GGATCCCATCATCATCATCATCATTAAAAGCT	2611 bp	1. 98°C, 0:30 min<br>2. 98°C, 0:10 min<br>3. 60°C, 0:30 min<br>4. 72°C, 0:55min<br>5. 72°C, 5:00 min<br>(Steps 2-4 × 34)
pHIS17 rv	CATATGTATATCTCCTTCTTAAAGTTAAACAAAATTATTTCTAGAGGGA		
A1_tei fw	GGAGATATACATATGAGTACGGTTCCTGAGCTGCT	1482 bp	1. 98°C, 0:30 min<br>2. 98°C, 0:10 min<br>3. 60°C, 0:30 min<br>4. 72°C, 0:30 min<br>5. 72°C, 2:00 min<br>(Steps 2-4 × 34)
A1_tei rv	ATGATGATGGGATCCATCGGCAGCGAATACCGG		
A1_tei fw	GGAGATATACATATGAGTACGGTTCCTGAGCTGCT	1186 bp	1. 98°C, 0:30 min<br>2. 98°C, 0:10 min<br>3. 60°C, 0:30 min<br>4. 72°C, 0:25 min<br>5. 72°C, 2:00 min<br>(Steps 2-4 × 34)
A1$_{tei-core}$_tcp9 rv	ATGATGATGGGATCCCTGGTCGTCCGCACGG		
A1$_{tei-core}$_graft fw	GCCACTTGGCACTTACTGGAAC	3481 bp	1. 98°C, 0:30 min<br>2. 98°C, 0:10 min<br>3. 60°C, 0:30 min<br>4. 72°C, 1:10min<br>5. 72°C, 5:00 min<br>(Steps 2-4 × 34)
A1$_{tei-core}$_graft rv	ATCAAACGCATACGGGGCATG		

Table S4. Comparison of the protein sequences (blastp) of the ristomycin NRPS with the reconstructed sequences.

Proteins *A. japonicum*	Proteins p3SV construct	Query cover %	% identity
AIG79240.1	NRPS4	98	84.93
AIG79241.1	NRPS3	99	79.21
AIG79242.1	NRPS2	98	76.93
AIG79243.1	NRPS1	100	76.11

Table S5. Comparison of amino acid selectivity codes for paleomycin with vancomycin, ristomycin and teicoplanin.

	A1	A2	A3	A4	A5	A6	A7
Van	DAFYLGMMCK (Leu)	DTSKVAAICK (Bht)	DLTKLGEVGK (Asn)	DIFHLGLLCK (Hpg)	DAVHLGLLCK (Hpg)	DASTLGAICK (Bht)	DPYHEGTLCK (Dpg)
Ris	DACHLGLLCK (Hpg)	DTSKTAAICK (Bht)	DPYNQGTFCK (Dpg)	DIFHLGLLCK (Hpg)	DAVHLGLLCK (Hpg)	DASTLGAICK (Bht)	DPYHEGTLCK (Dpg)
Tei	DAFHLGLLCK (Hpg)	DASTVAAVCK (Tyr)	DAYNLGTLCK (Dpg)	DIFHLGLLCK (Hpg)	DALHLGLLCK (Hpg)	DASTIAGVCK (Bht)	DPYHGGTLCK (Dpg)
Paleo	DAFHLGLLCK (Hpg)	DASTVAAVCK (Tyr)	DAYNPGTLCK (Dpg)	DIFHLGLLCK (Hpg)	DALHLGLLCK (Hpg)	DASTVAAVCK (Tyr)	DPYHGGTLCK (Dpg)

Table S6. X-Ray Data Collection and Refinement Statistics. Values in parentheses are for highest resolution shell.

Protein	**8GJ4** - Tcp9 A1$_{core\text{-}tei}$ (apo)	**8GIC** - Tcp9 A1$_{core\text{-}tei}$ (Hpg)	**8GJP** - Tcp9 A1$_{core\text{-}ANC2}$	**8GKM** - Tcp9 A1$_{core\text{-}ANC3}$	**8GLC** - ANC4$_{core}$
**Data collection**					
Space group	$P\ 2\ 2_1\ 2_1$	$P\ 2\ 2_1\ 2_1$	$P\ 2\ 2_1\ 2_1$	$P\ 2\ 2_1\ 2_1$	$I\ 4_1\ 3\ 2$
Unit-cell parameters (Å, °)	42.229 123.997 176.2 90 90 90	42.218 123.794 176.587 90 90 90	42.747 123.419 175.789 90 90 90	41.805 123.415 175.171 90 90 90	261.925 261.925 261.925 90 90 90
Resolution range (Å)	44.05 - 1.805 (1.87 - 1.805)	44.15 - 1.637 (1.696 - 1.637)	43.95 - 2.696 (2.792 - 2.696)	43.79 - 1.887 (1.954 - 1.887)	47.82 - 3.124 (3.236 - 3.124)
Wavelength	0.9537	0.9537	0.9536	0.9536	0.9536
Total reflections	473300 (43849)	783214 (75621)	178473 (17136)	1002760 (96102)	246347 (23851)
Unique reflections	84677 (7822)	114850 (11071)	26545 (2529)	74127 (7224)	27303 (2637)
Completeness (%)	98.11 (91.97)	99.71 (97.24)	99.46 (95.45)	99.49 (95.35)	99.39 (97.99)
Rpim† (%)	4.21 (38.68)	2.81 (42.73)	12.02 (90.29)	6.28 (110.8)	3.115 (20.97)
(I/σ(I))	13.46 (2.45)	14.03 (1.87)	5.60 (0.91)	9.48 (1.03)	14.69 (3.38)
$CC_{1/2}$†† (%)	99.8 (83.8)	99.6 (72.4)	99 (41.7)	99.8 (38.5)	99.8 (84.6)
CC*††† (%)	100 (95.5)	99.9 (91.6)	99.7 (76.7)	100 (74.6)	100 (95.7)
Rmerge‡ (%)	9.2 (85.77)	6.822 (105.1)	28.84 (218.8)	22.37 (394.2)	9.079 (59.68)
Average multiplicity	5.6 (5.6)	6.8 (6.8)	6.7 (6.8)	13.5 (13.3)	9.0 (8.9)
Wilson B-factor	24.09	21.45	51.16	28.7	97.31
**Structure refinement**					
R_{work}/R_{free}‡‡ (%)	18.57 / 21.03	16.61 / 19.73	22.12 / 25.97	18.90 / 21.93	22.89 / 26.68
Rmsd, bond lengths (Å)	0.006	0.006	0.004	0.004	0.004
Rmsd, bond angles (°)	0.99	0.97	0.91	0.75	0.68
Ramachandran angles §					
Favoured	98.30	98.22	98.3	98.63	99.03
Allowed	1.70	1.78	1.59	137	0.97
Outliers	0	0	0.11	0	0
B-factors					
Average B-factor	29.55	27.13	54.55	36.07	88.44
ligands	41.95	47.62	-	47.94	-
solvent	33.80	37.31	-	38.06	-

†$R_{p.i.m.} = \sum_{hkl}\{1/[N(hkl) - 1]\}^{1/2} \times \sum_i |I_i(hkl) - \langle I(hkl)\rangle| \sum_{hkl} \sum_i I_i(hkl)$
††$CC_{1/2} = \sum(x - \langle x\rangle)(y - \langle y\rangle)/[\sum(x - \langle x\rangle)^2 \sum(y - \langle y\rangle)^2]^{1/2}$
†††$CC* = \sqrt{\frac{2CC_{1/2}}{1+CC_{1/2}}}$
‡ $R_{merge} = \sum_{hkl} \sum_i |I_i(hkl) - \langle I(hkl)\rangle| / \sum_{hkl} \sum_i I_i(hkl)$
‡‡$R = \frac{\sum ||F_{obs}| - |F_{calc}||}{\sum |F_{obs}|}$
§ Categories were defined by MolProbity[9]

Supplementary Figures

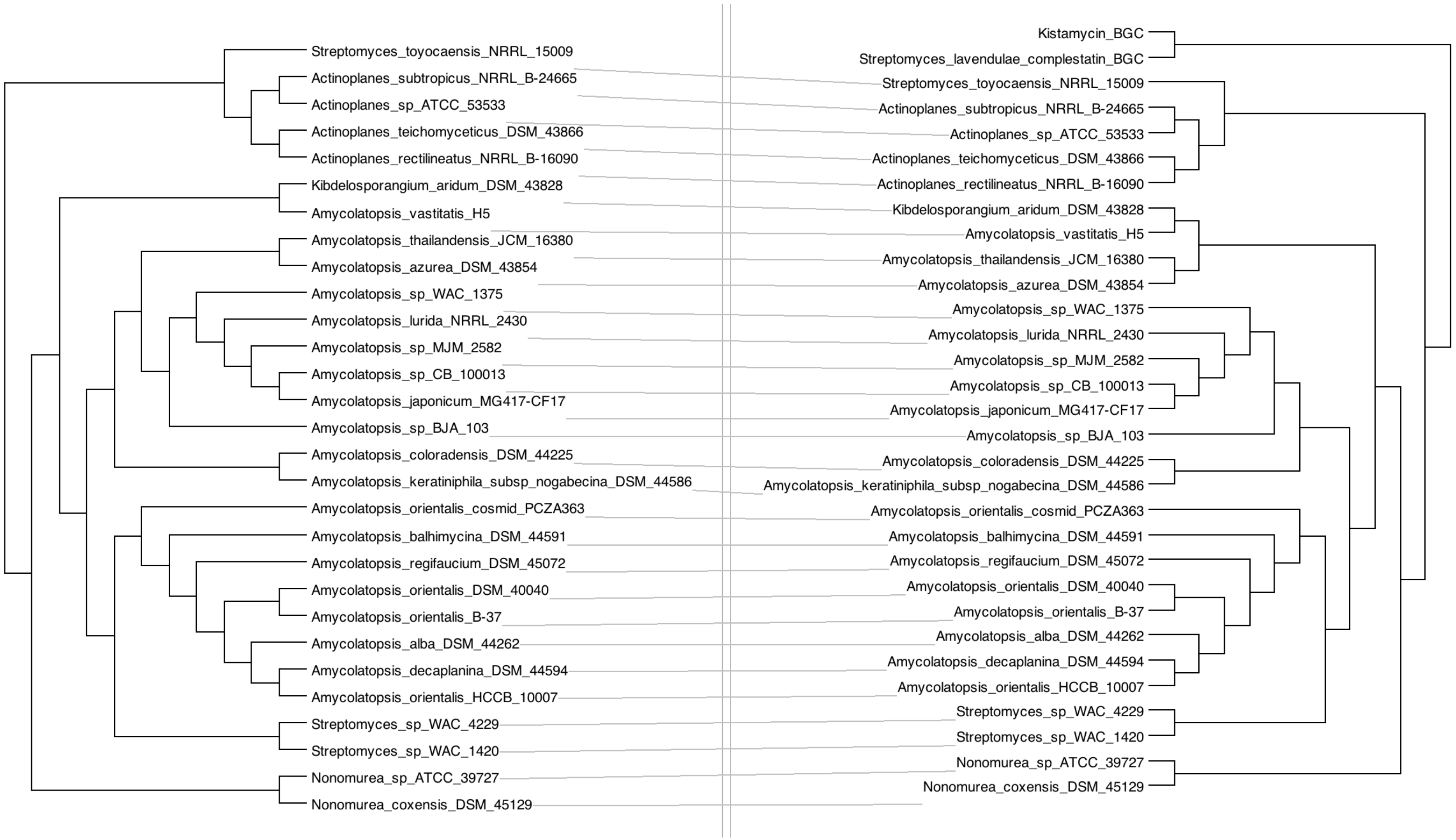

Figure S1: Tanglegram visualising the congruence of a midpoint rooted guide tree (left panel) vs. an outgroup rooted guide tree (right panel). The tanglegram was computed using Dendroscope 3.[10]

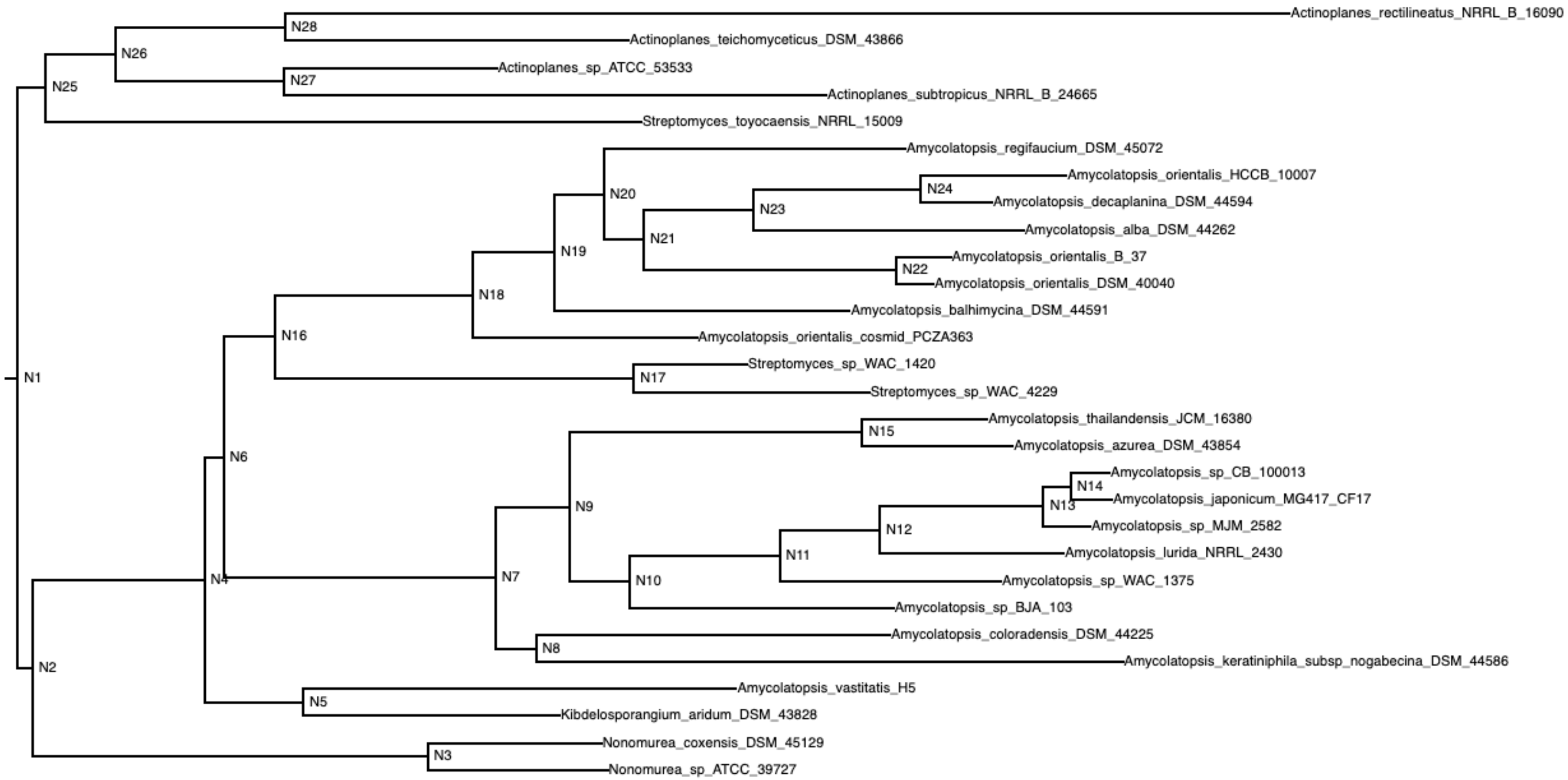

Figure S2: Concatenated NRPS tree used as guide tree for ancestral sequence reconstruction and ancestral state reconstruction. Reconstruction at the root node N1 was used for paleomycin sequence reconstruction. Tree was visualised in IcyTree.[11]

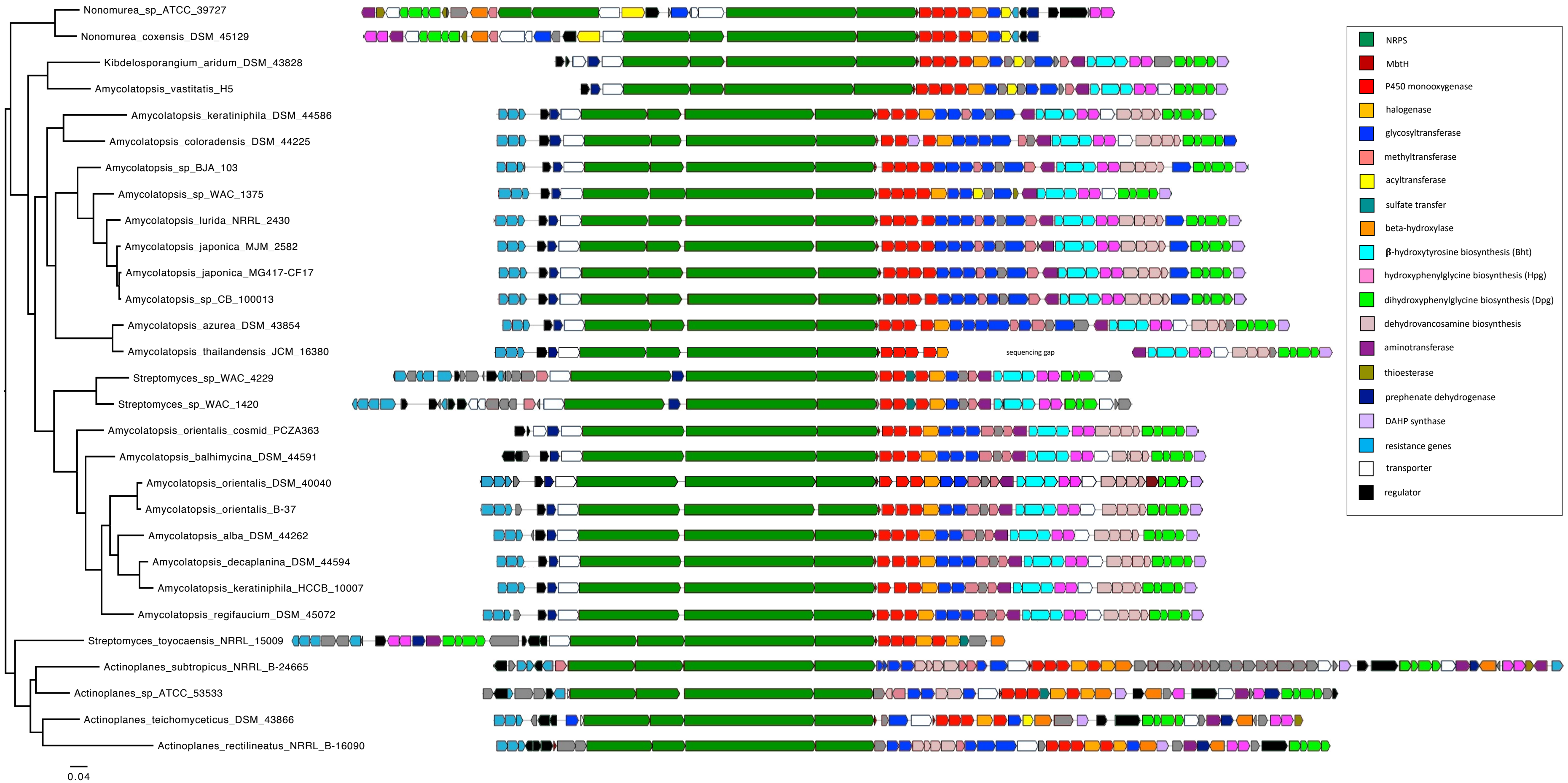

Figure S3: Overview of the phylogeny and cluster architecture of the GPA gene clusters. The midpoint rooted maximum likelihood phylogenetic tree is based on the concatenation of all NRPS sequences.

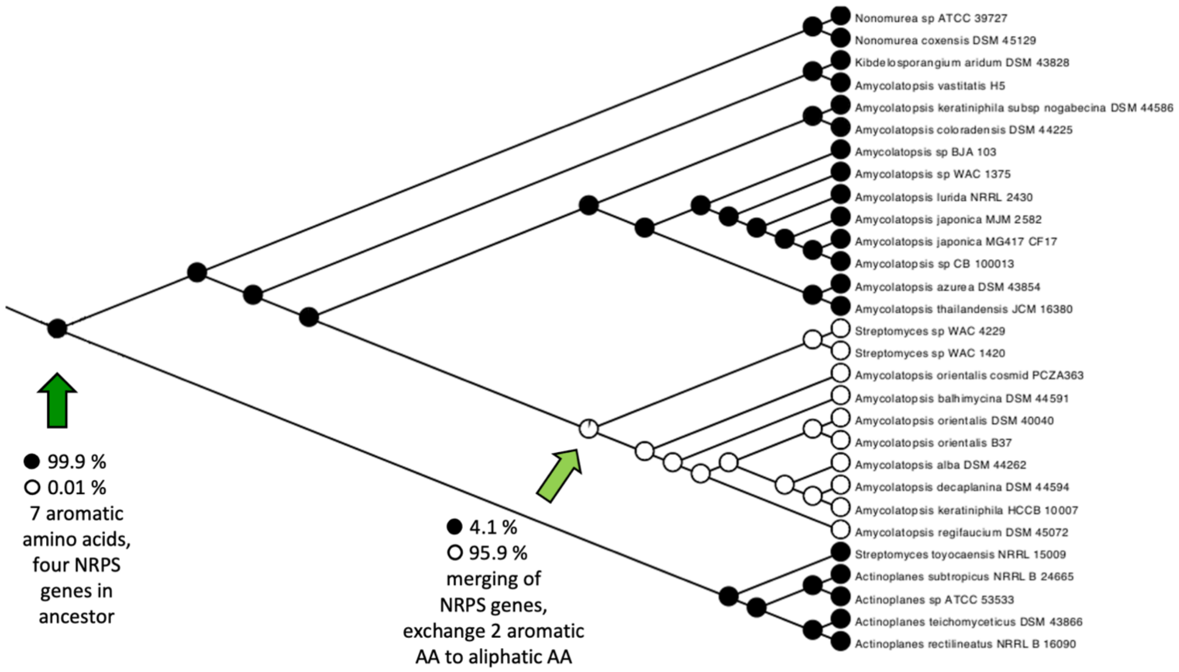

Figure S4: Ancestral state reconstruction for GPA NRPS genes. Phylogenetic traits: (○) white = three NRPS genes with building blocks incorporating five aromatic amino acids and two aliphatic amino acids; (●) black - four NRPS genes with building blocks incorporating 7 aromatic amino acids.

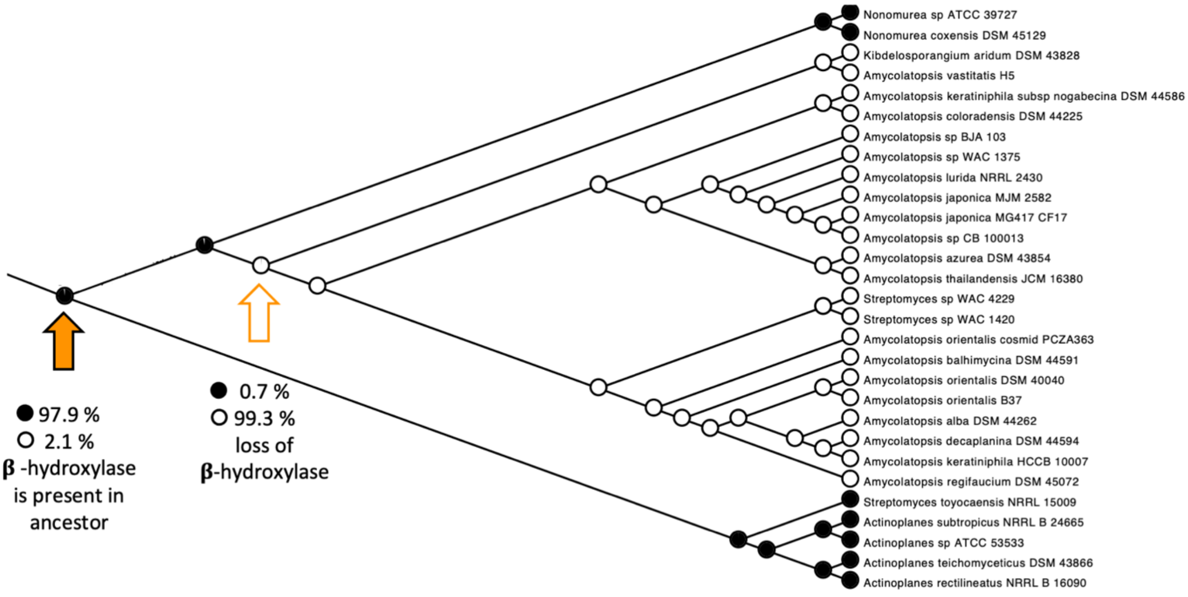

Figure S5: Ancestral state reconstruction for the β-hydroxylase gene. Phylogenetic traits: (○) white = β-hydroxylase gene absent; (●) black - β-hydroxylase gene present.

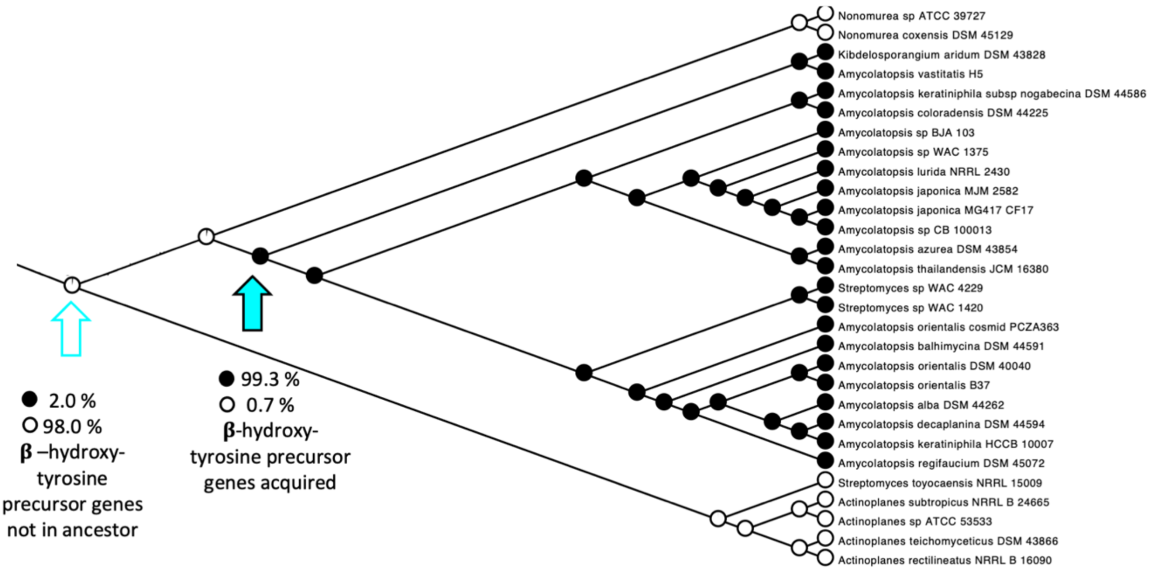

Figure S6: Ancestral state reconstruction for the β-hydroxytyrosine subcluster. Phylogenetic traits: (○) white = β-hydroxytyrosine subcluster absent; (●) black - β-hydroxytyrosine subcluster present.

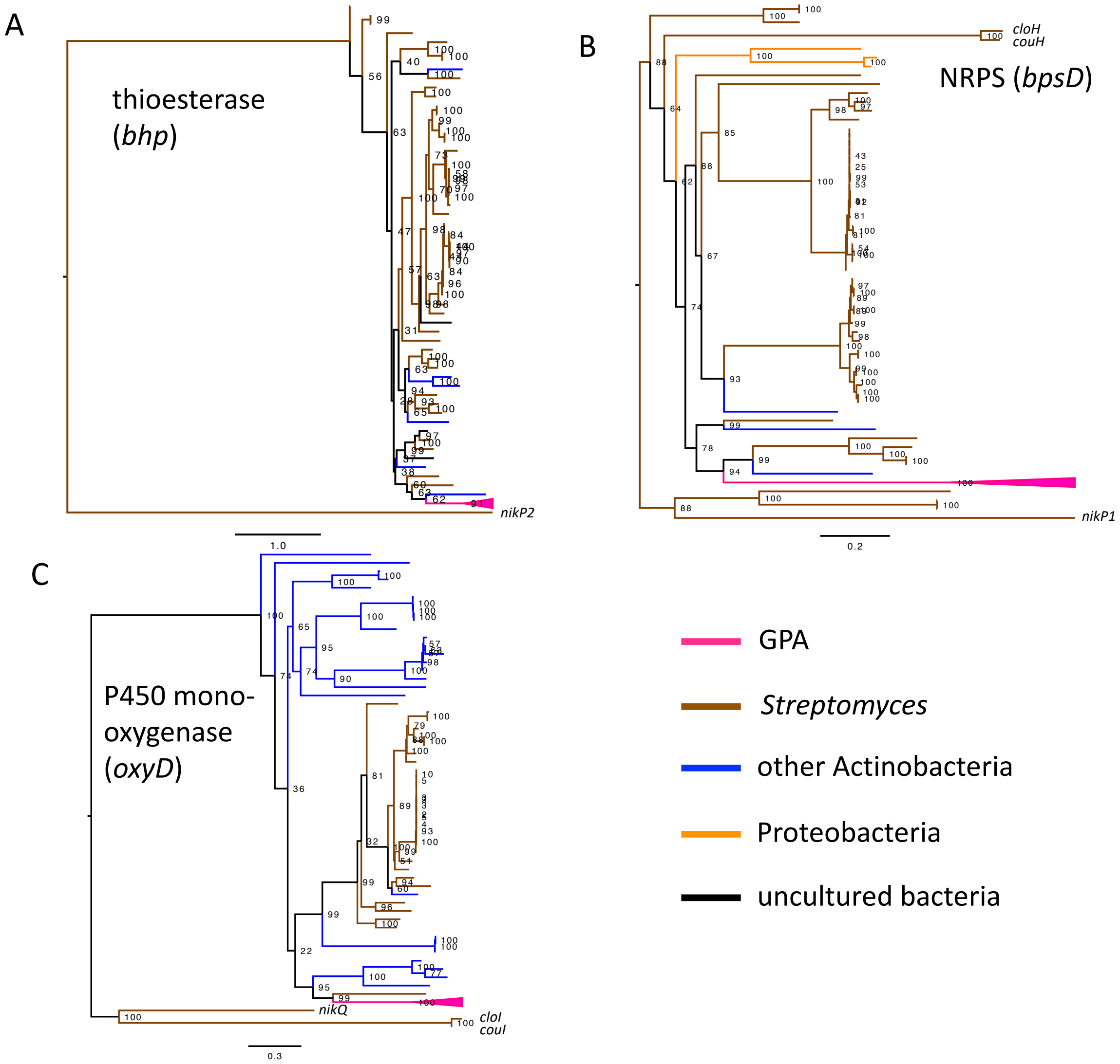

Figure S7: Maximum likelihood trees for β-hydroxytyrosine (Bht) biosynthesis genes. Phylogenetic trees are based on an alignment of nearest blast hits, using the *A. japonica* MG417-CF17 genes as query. Representative for genes with a similar mechanism, β-hydroxyhistidine biosynthesis genes from the nikkomycin BGC as well as Bht biosynthesis genes from the clorobiocin and coumermycin BGCs are included. **A** thioesterase gene (*bhp*) homologs, **B** NRPS gene (*bspD*) homologs and **C** the P450 monooxygenase gene (*oxyD*) homologs. Sequences were aligned using the MAFFT E-INS-i algorithm (default parameters). Phylogenetic trees were calculated with IQtree, using the LG+F+I+G4 model.

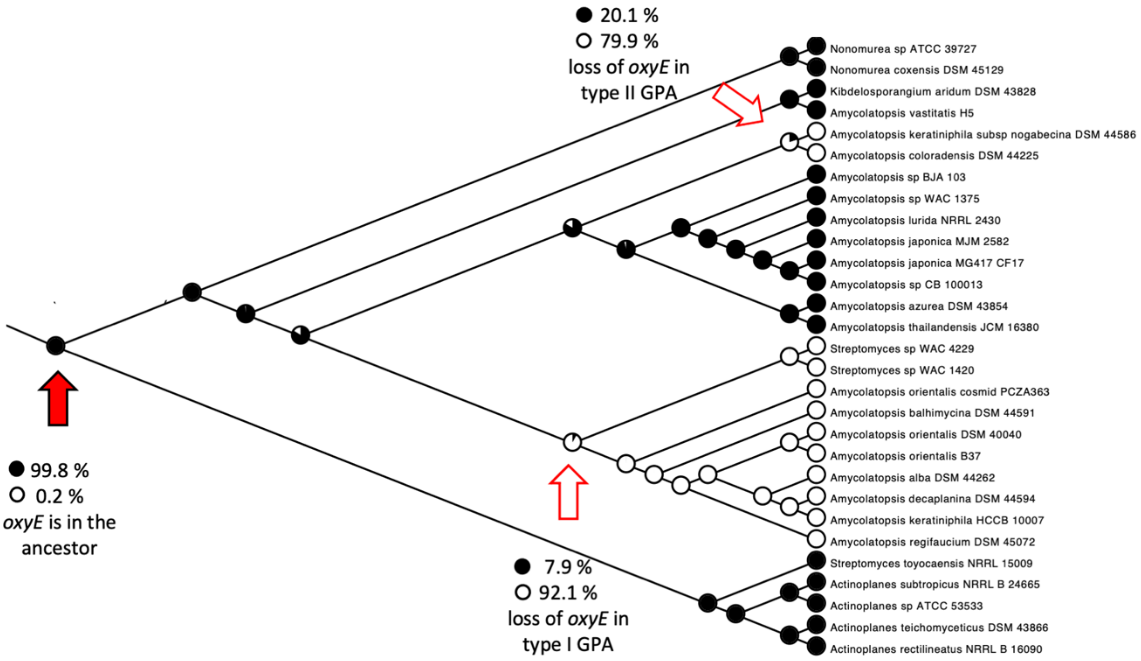

Figure S8: Ancestral state reconstruction for the P450 monooxygenase *oxyE*. Phylogenetic traits: (○) white = *oxyE* absent; (●) black - *oxyE* present.

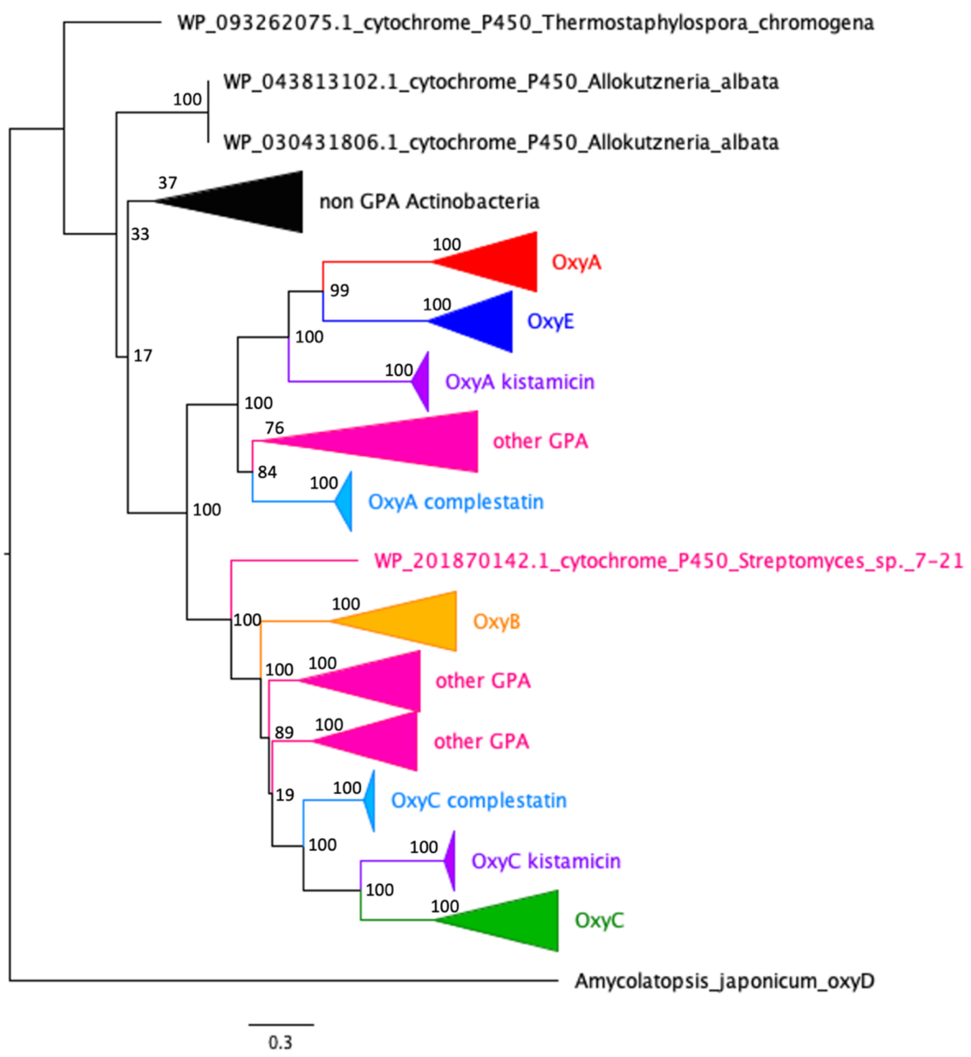

Figure S9: Maximum likelihood tree of GPA P450 monooxygenases with crosslinking activity and their most closely related homologs. OxyD was used as an outgroup. Sequences were aligned using the MAFFT E-INS-i algorithm (default parameters). Phylogenetic tree was calculated with IQtree, using the LG+F+R6 model.

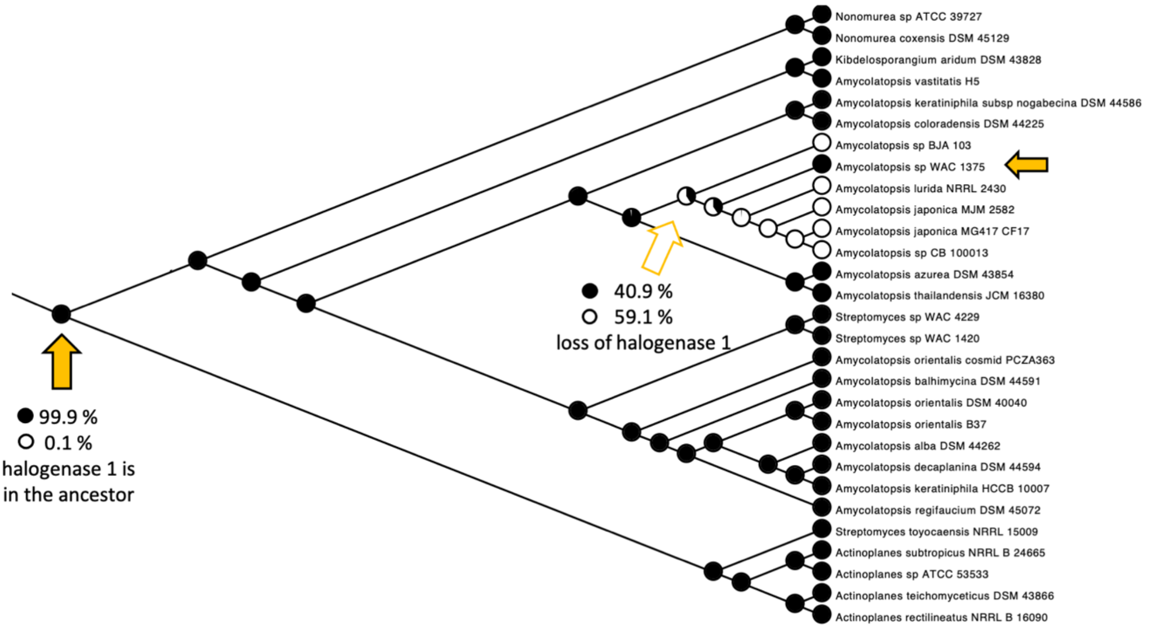

Figure S10: Ancestral state reconstruction for halogenase BhaA. Phylogenetic traits: (○) white = BhaA absent; (●) black – BhaA present.

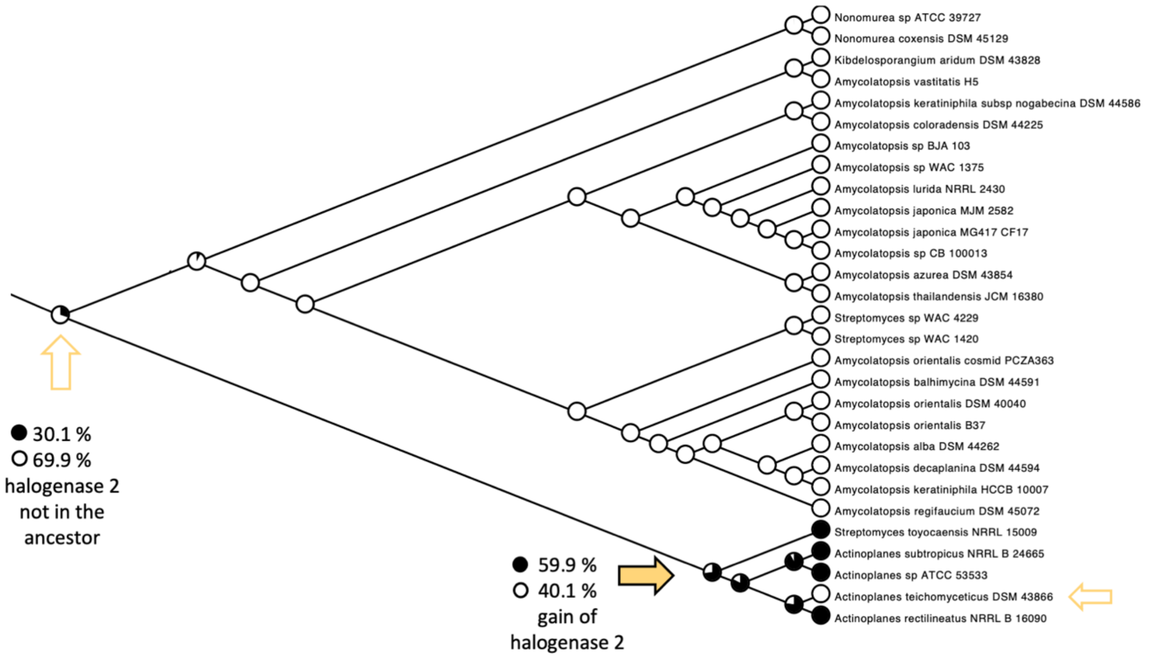

Figure S11: Ancestral state reconstruction for halogenase *staK*. Phylogenetic traits: (○) white = *staK* absent; (●) black – *staK* present.

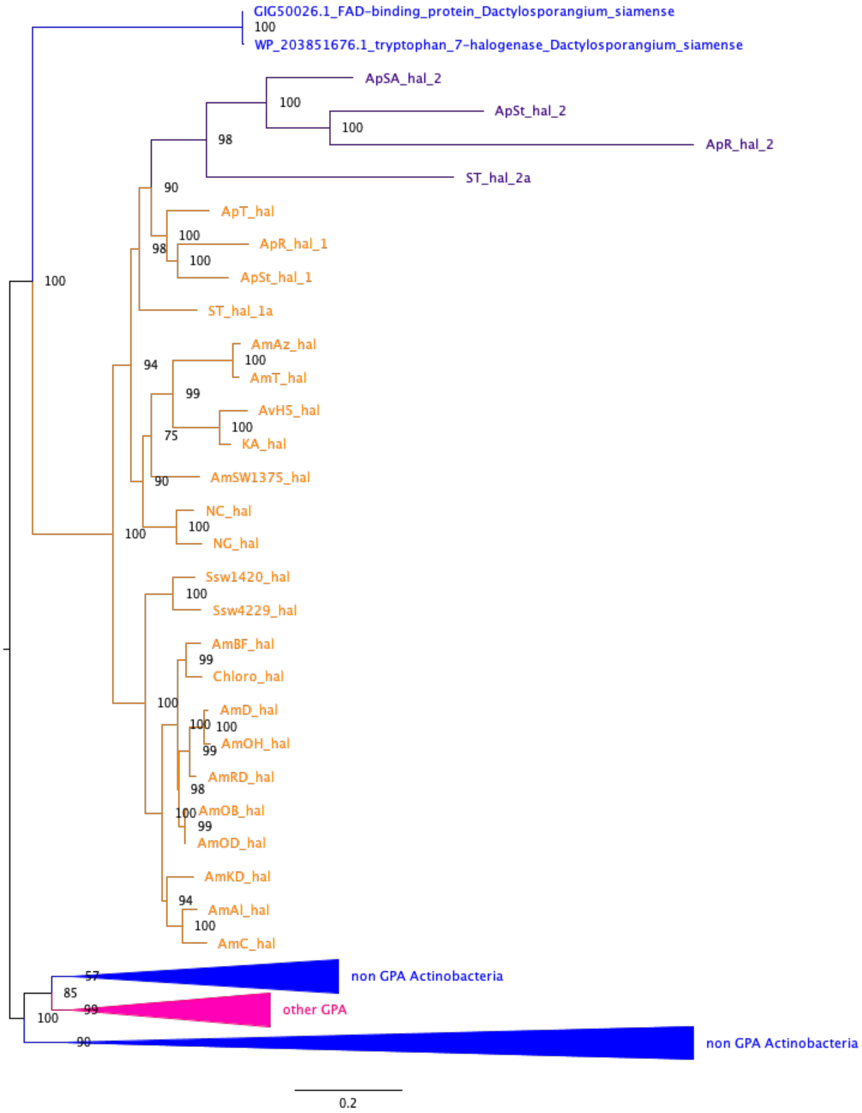

Figure S12: Maximum likelihood tree of GPA halogenases with their most closely related homologs. StaK-like halogenases are shown in orange, BhaA-like halogenases are shown in purple. The tree was midpoint rooted. Sequences were aligned using the MAFFT E-INS-i algorithm (default parameters). Phylogenetic tree was calculated with IQtree, using the LG+F+R7 model.

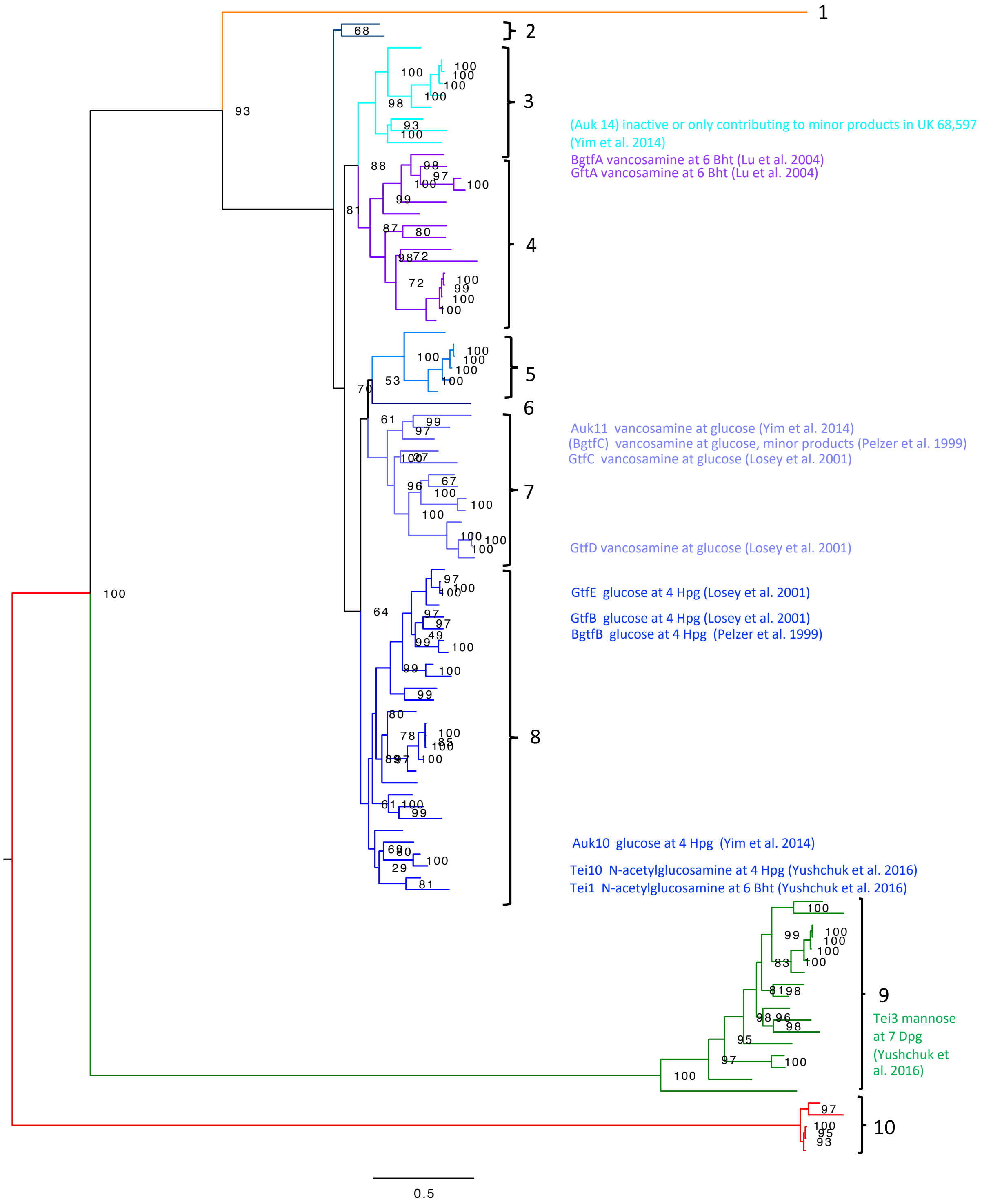

Figure S13: Maximum likelihood tree of GPA glycosyltransferases. Tree was midpoint rooted. Sequences were aligned using the MAFFT E-INS-i algorithm (default parameters). Phylogenetic tree was calculated with IQtree, using the JTT+F+G4 model. Phylogenetic subgroups (1-10) were assigned based on the functions reported from literature.

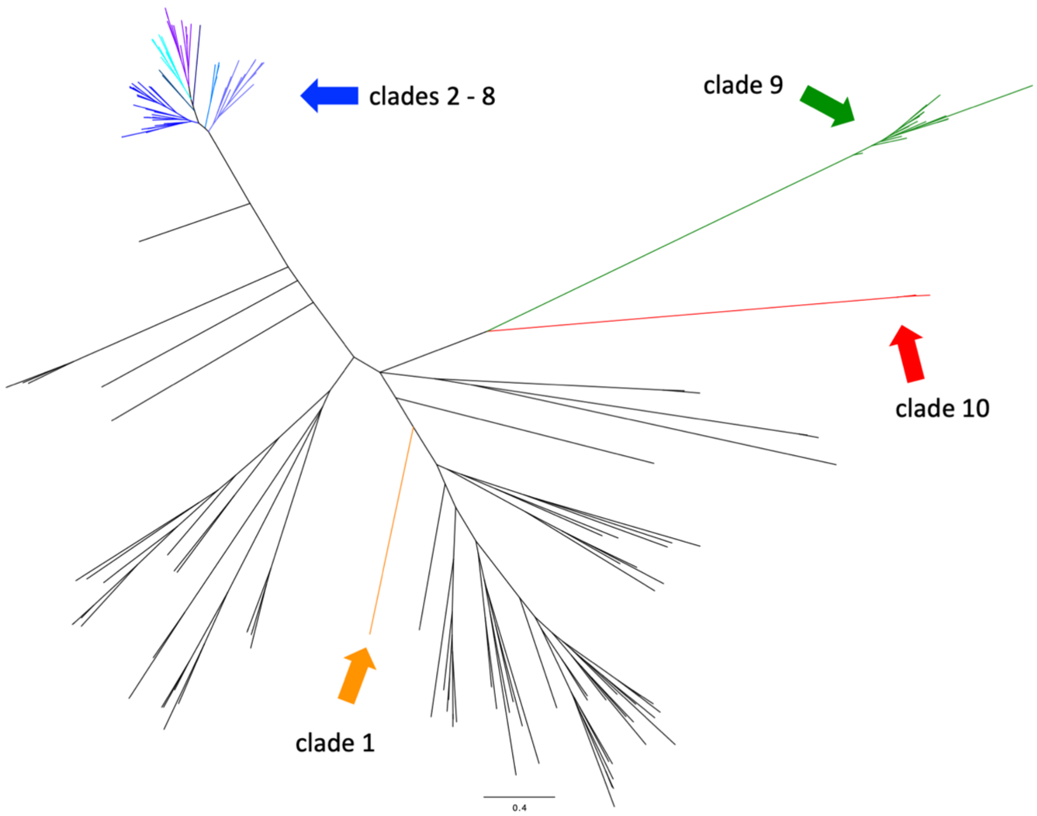

Figure S14: Maximum likelihood tree of GPA glycosyltransferases. Sequences were aligned using the MAFFT E-INS-i algorithm (default parameters). Phylogenetic tree was calculated with IQtree, using the WAG+F+R6 model. Phylogenetic subgroups (1-10) were aligned with CAZy database GT1 group genes with characterised function.

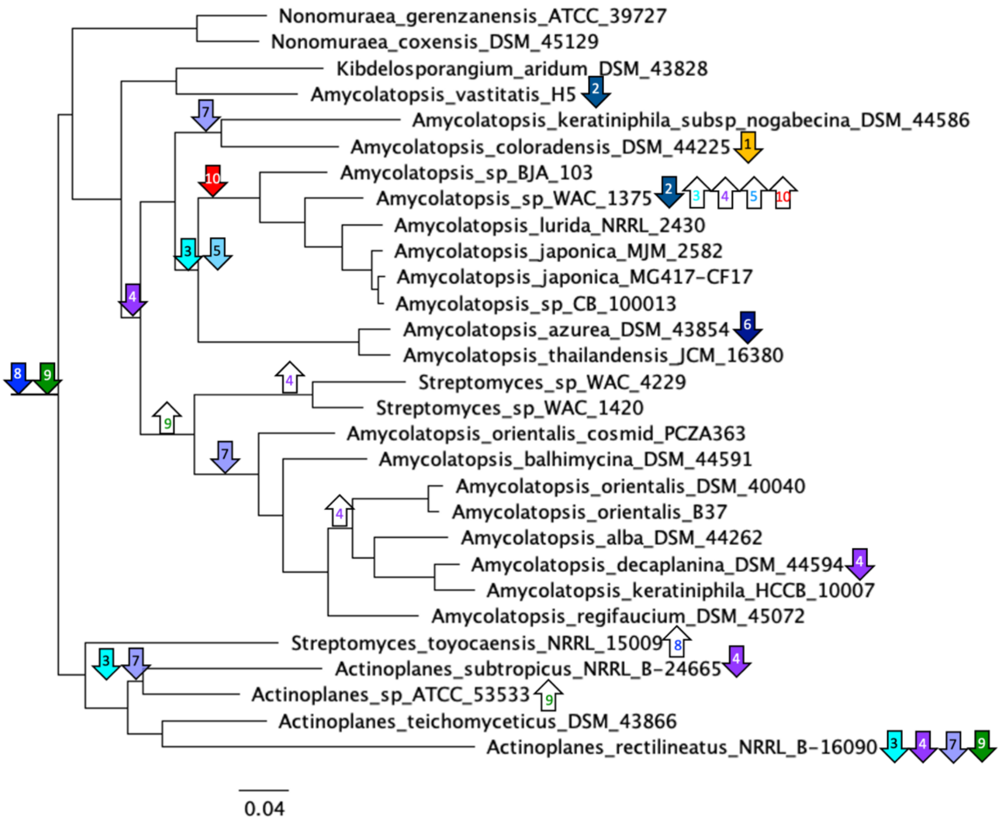

Figure S15: GPA evolution guide tree, indicating gain or loss of glycosyltransferases. Numbers are referring to phylogenetic groups as shown in Fig S11. Downward facing, fully colored arrows are indicating gain of glycosyltransferases. Upward facing, white arrows are indicating loss of glycosyltransferases. Arrows are representative for the respective phylogenetic clade when assigned to a branch of the tree, or to a single strain when assigned to the tip labels.

A

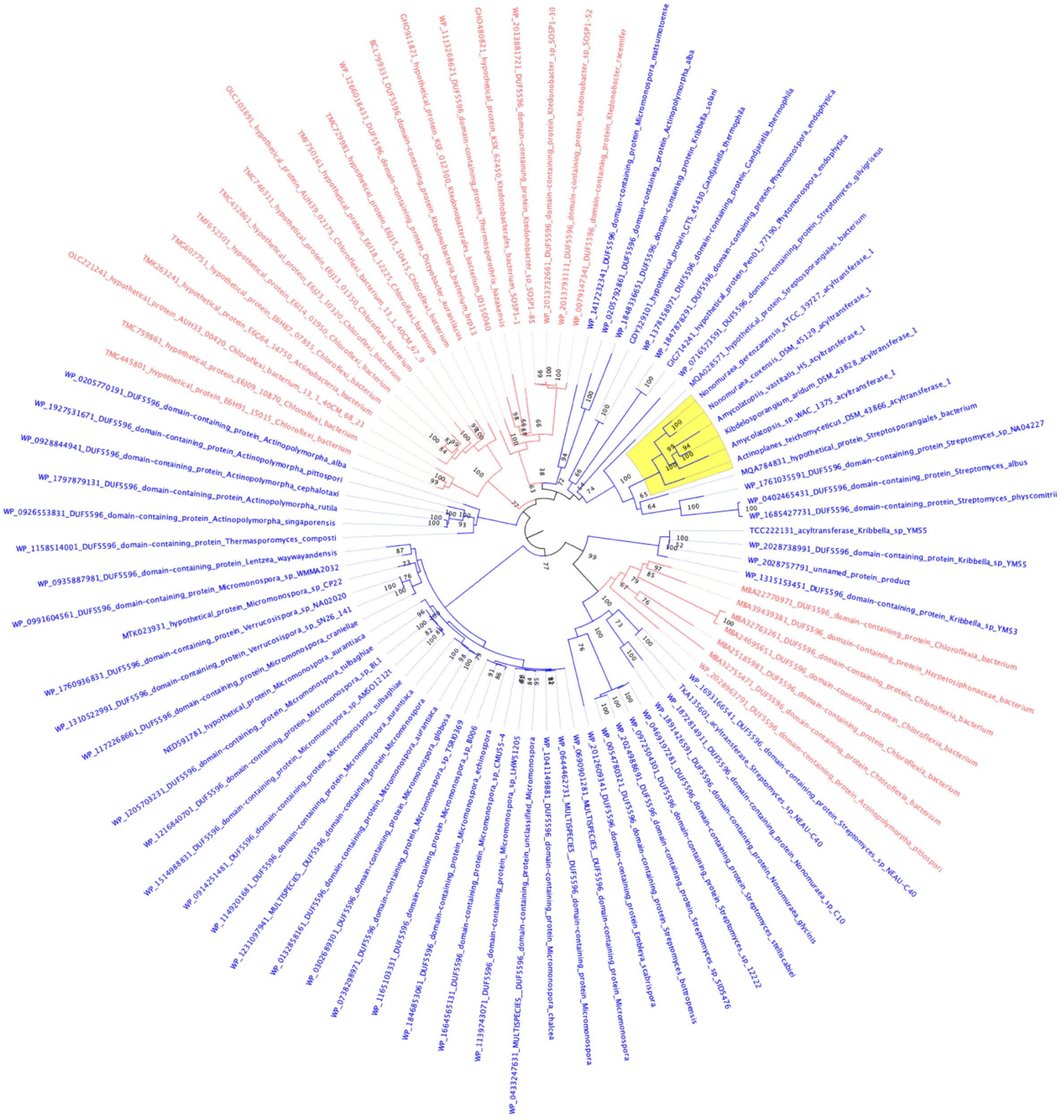

Figure S16 A: Maximum likelihood tree of GPA acyltransferases and their most closely related homologs. A) Tei11/Dbv8-like N-acyltransferases (highlighted in yellow) and their closest homologs according to blast similarity. Tree was midpoint rooted. Sequences were aligned using the MAFFT E-INS-i algorithm (default parameters). Phylogenetic tree was calculated with IQtree, using the LG+F+I+G4 model.

B

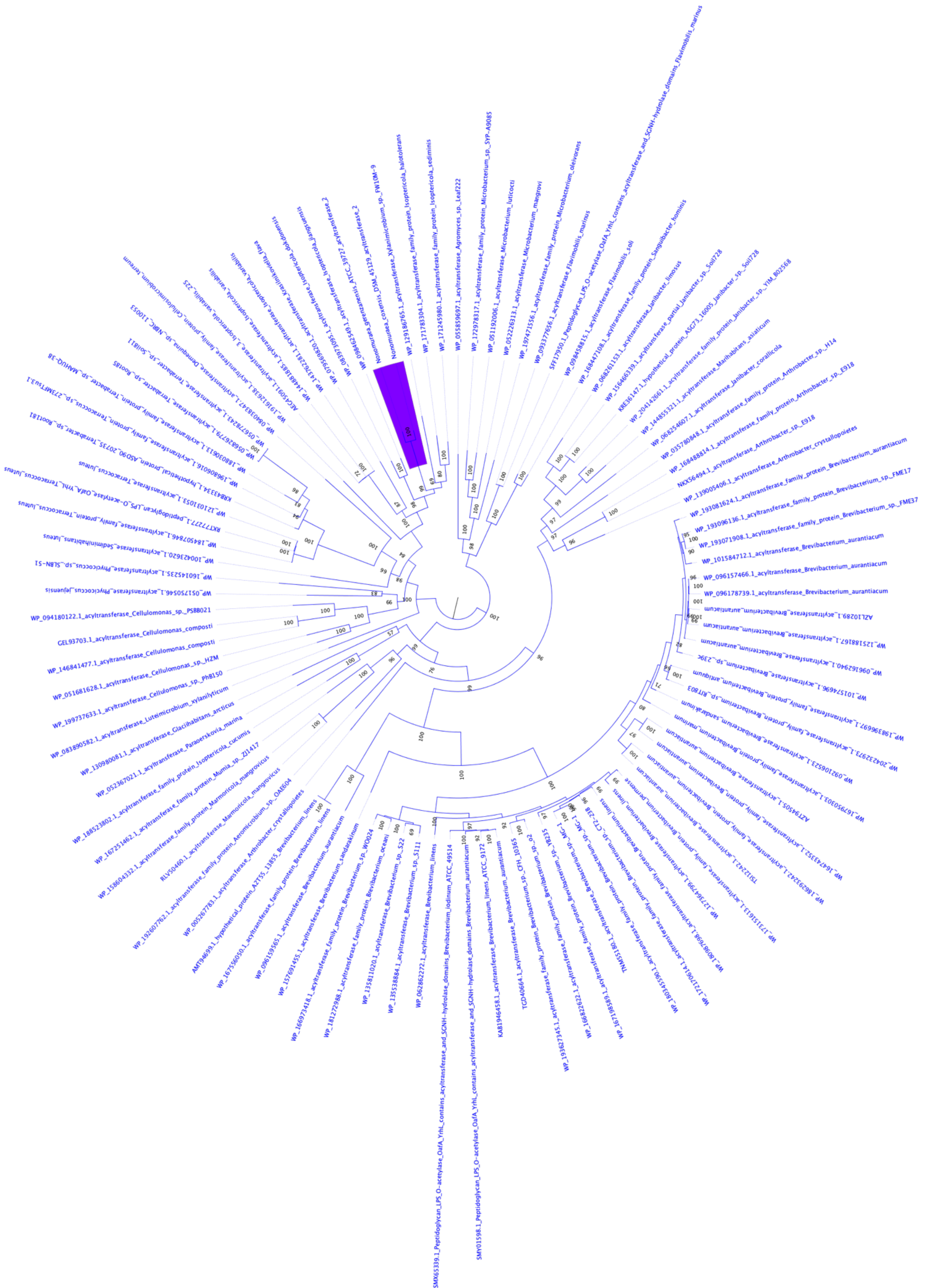

Figure S16 B: Maximum likelihood tree of GPA acyltransferases and their most closely related homologs. B) Dbv23-like O-acyltransferases (highlighted purple) and their closest homologs according to blast similarity. Trees were midpoint rooted. Sequences were aligned using the

MAFFT E-INS-i algorithm (default parameters). Phylogenetic tree was calculated with IQtree, using the WAG+F+R5 model.

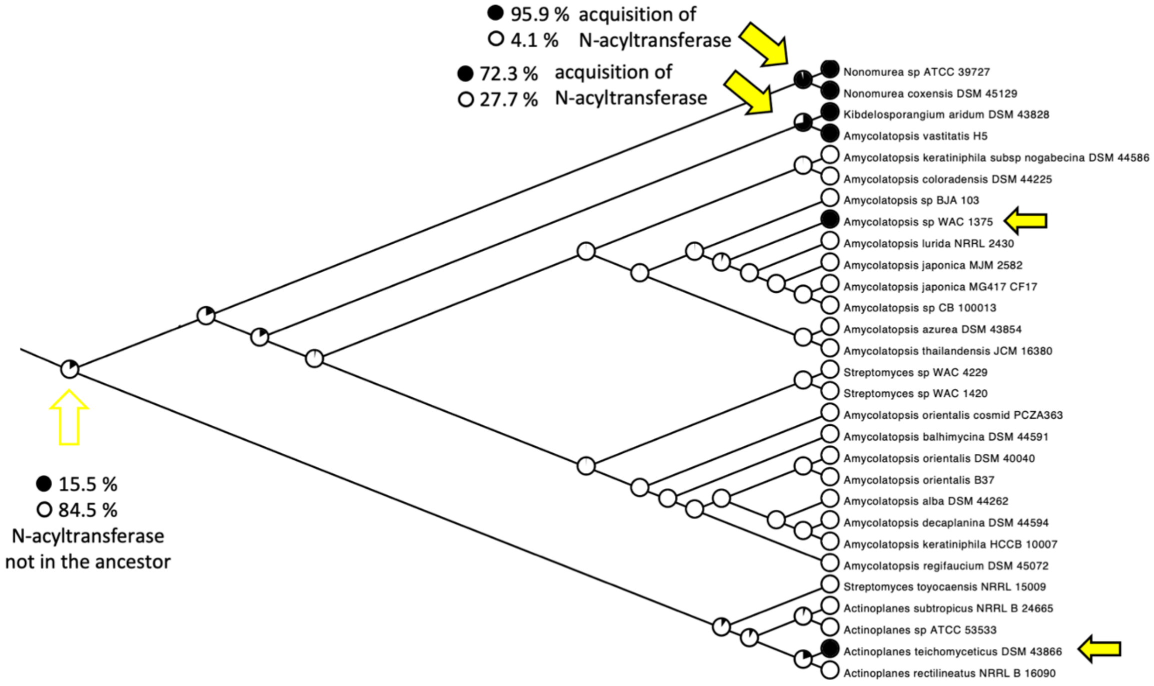

Figure S17: Ancestral state reconstruction for N-acyltransferases *dbv8*. Phylogenetic traits: (○) white = *dbv8* absent; (●) black – *dbv8* present.

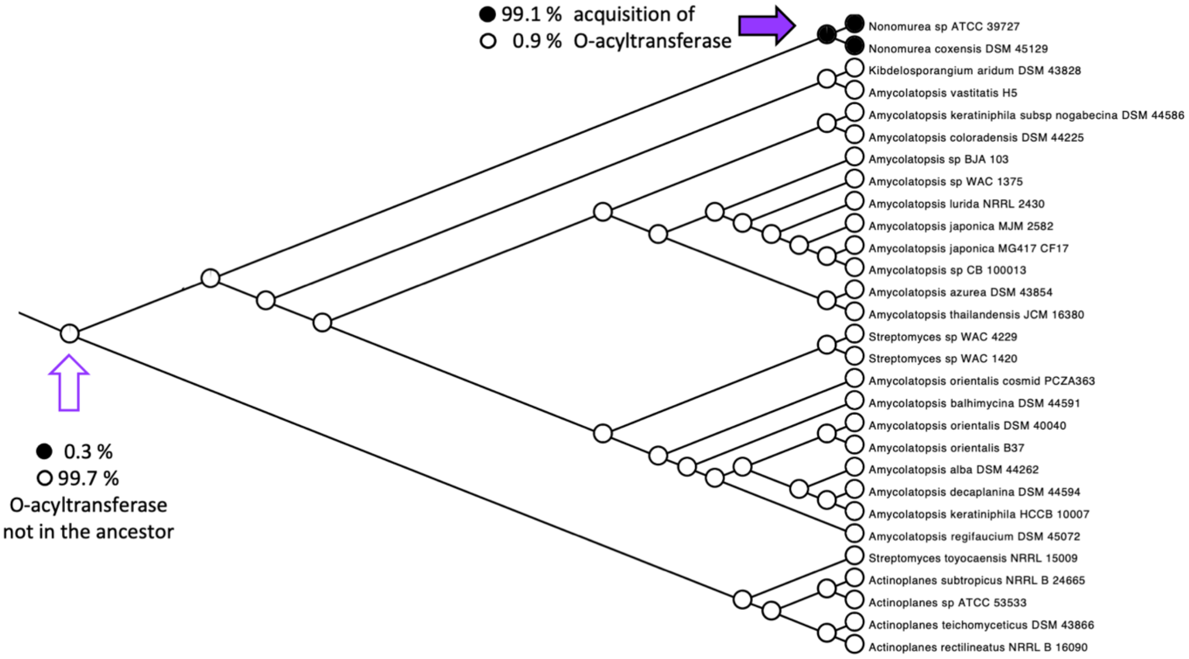

Figure S18: Ancestral state reconstruction for O-acyltransferases *dbv23*. Phylogenetic traits: (○) white = *dbv23* absent; (●) black – *dbv8* present.

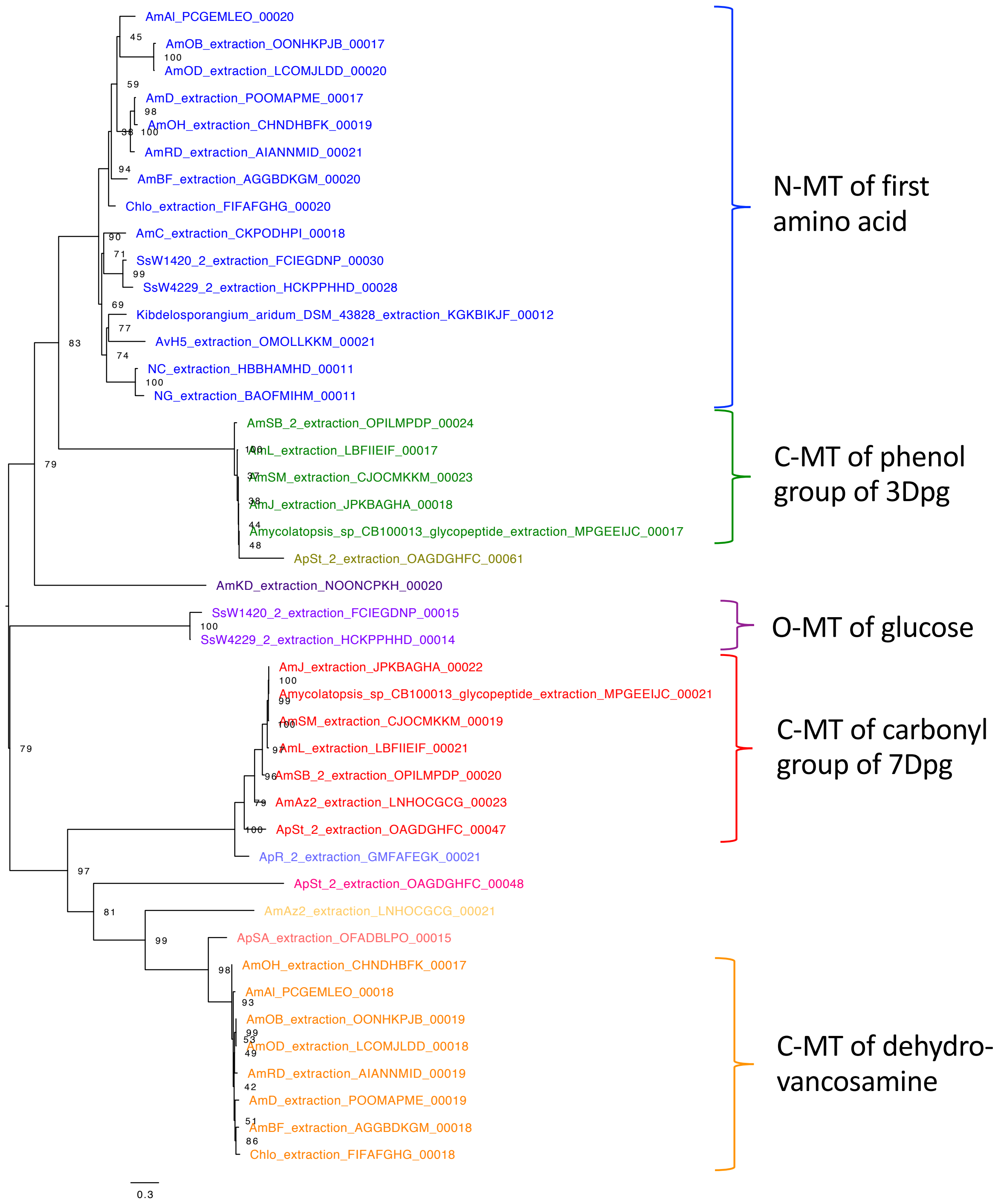

Figure S19: Maximum likelihood tree of GPA methyltransferases (MT). Tree was midpoint rooted. Sequences were aligned using the MAFFT E-INS-i algorithm (default parameters). Phylogenetic tree was calculated with IQtree, using the WAG+F+I+G4 model.

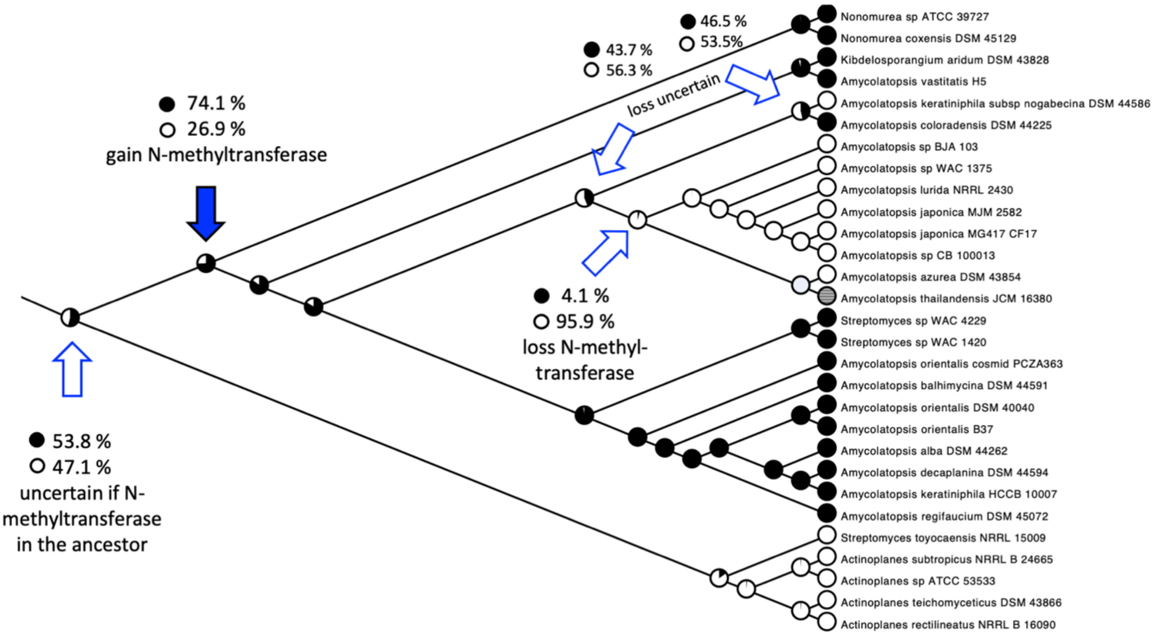

Figure S20: Ancestral state reconstruction for the *mtfA*-like 3Dpg methylating N-methyltransferase. Phylogenetic traits: (○) white = absent; (●) black – present.

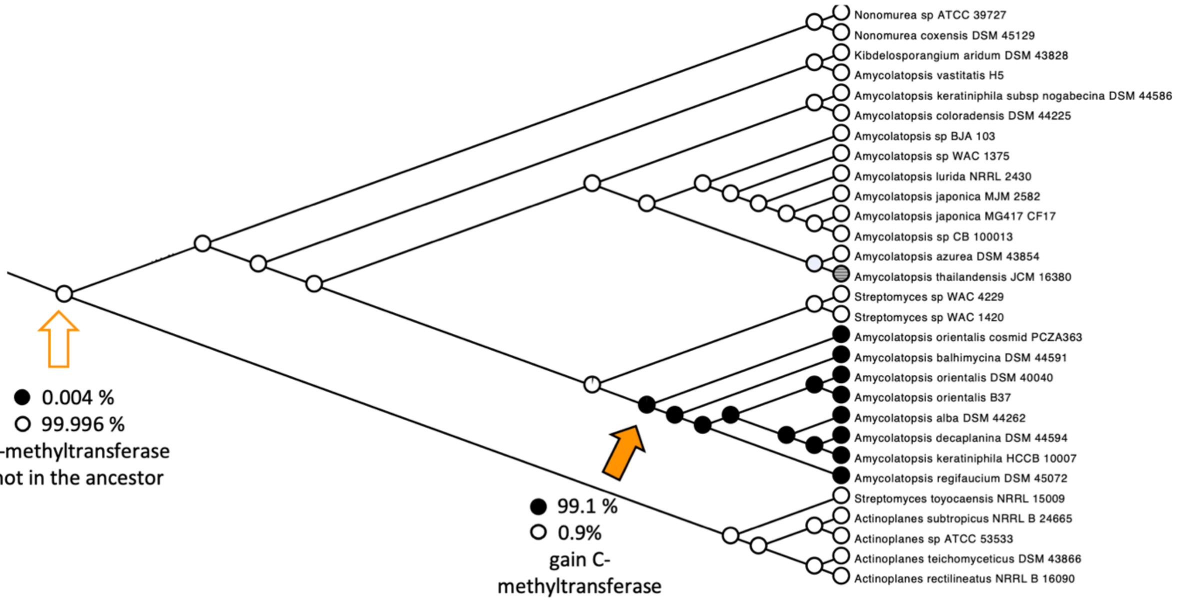

Figure S21: Ancestral state reconstruction for the *evaC*-like dehydrovancosamine methylating N-methyltransferase. Phylogenetic traits: (○) white = absent; (●) black – present.

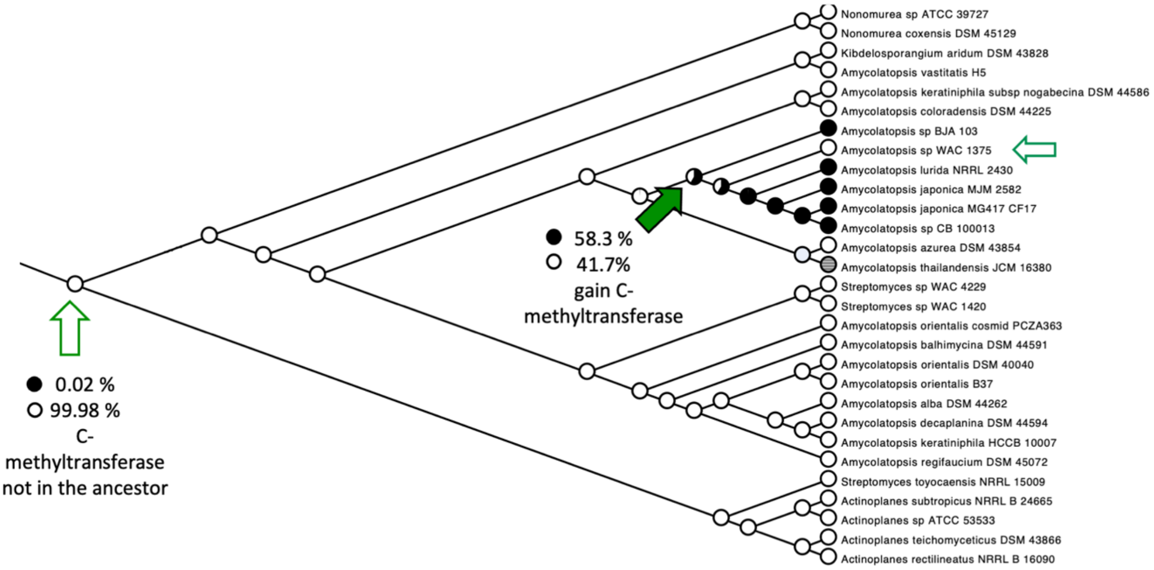

Figure S22: Ancestral state reconstruction for the phenol methylating C-methyltransferase. Phylogenetic traits: (○) white = absent; (●) black – present.

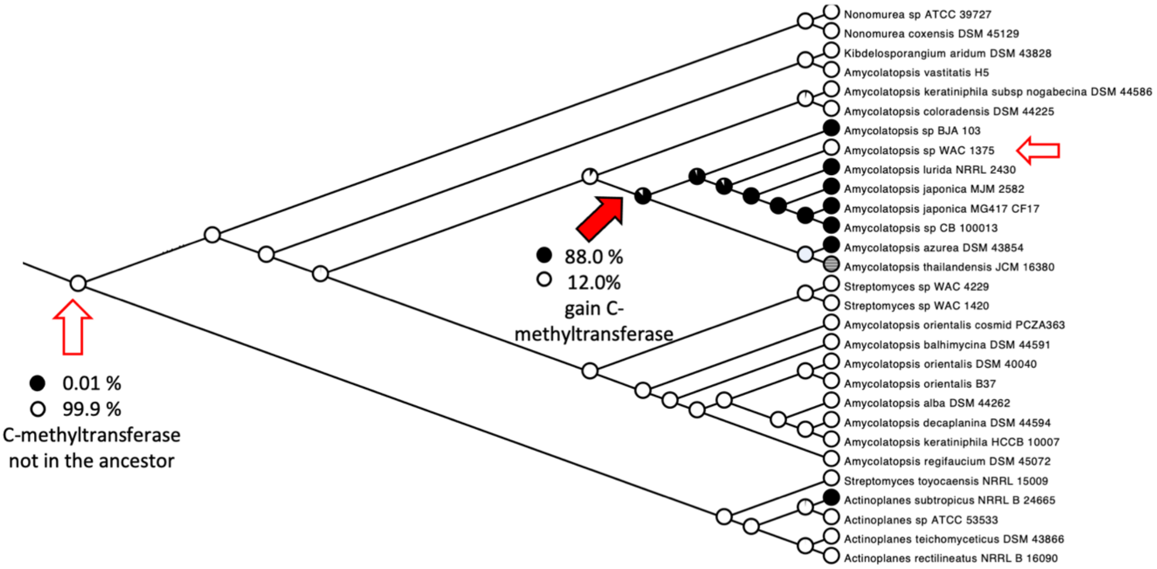

Figure S23: Ancestral state reconstruction for the 7Dpg methylating C-methyltransferase. Phylogenetic traits: (○) white = absent; (●) black – present.

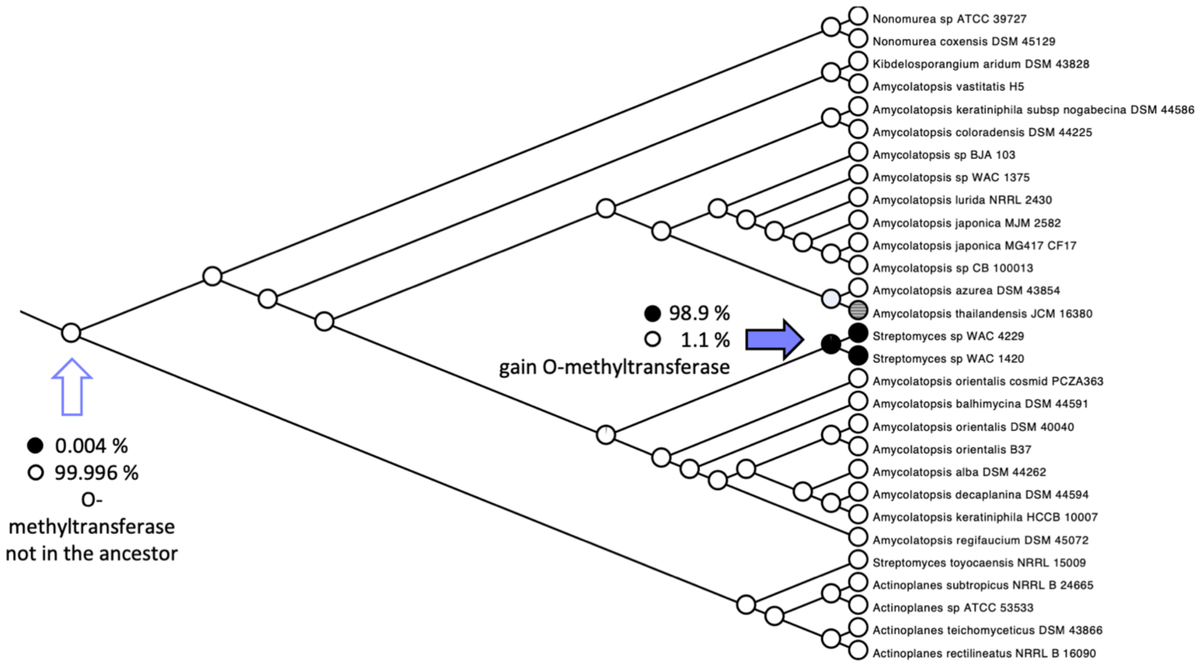

Figure S24: Ancestral state reconstruction for the O-methyltransferase. Phylogenetic traits: (○) white = absent; (●) black – present.

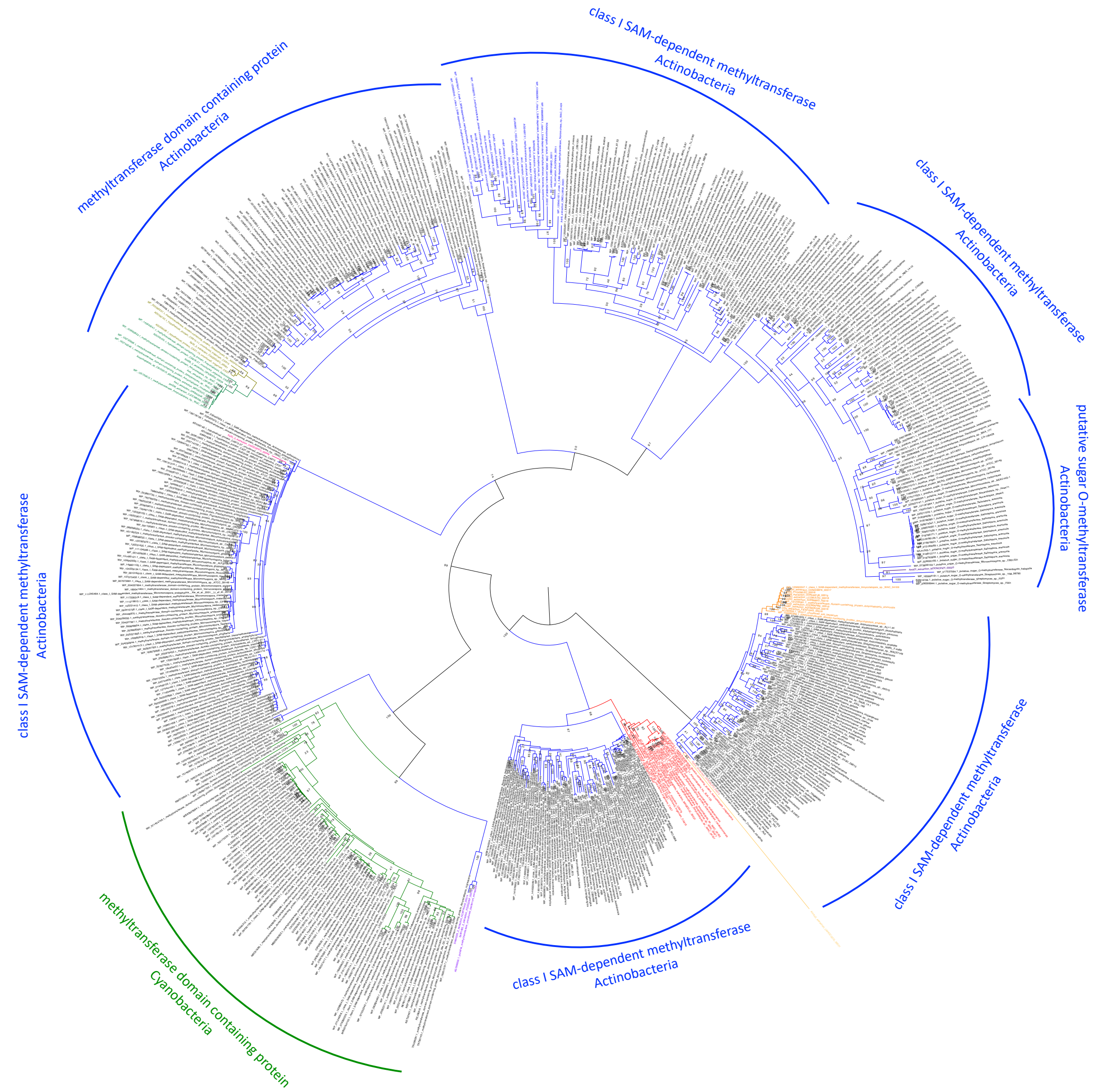

Figure S25: Maximum likelihood tree of methyltransferases (MT). Tree leaves are color coded according to the phylogenetic groups from **Figure S19**. Branch colouring: blue = Actinobacteria, green = cyanobacteria, GPA methyltransferases = subgroups from Fig S17. Tree was midpoint rooted. Sequences were aligned using the MAFFT E-INS-i algorithm (default parameters). Phylogenetic tree was calculated with IQtree, using the WAG+F+R7 model.

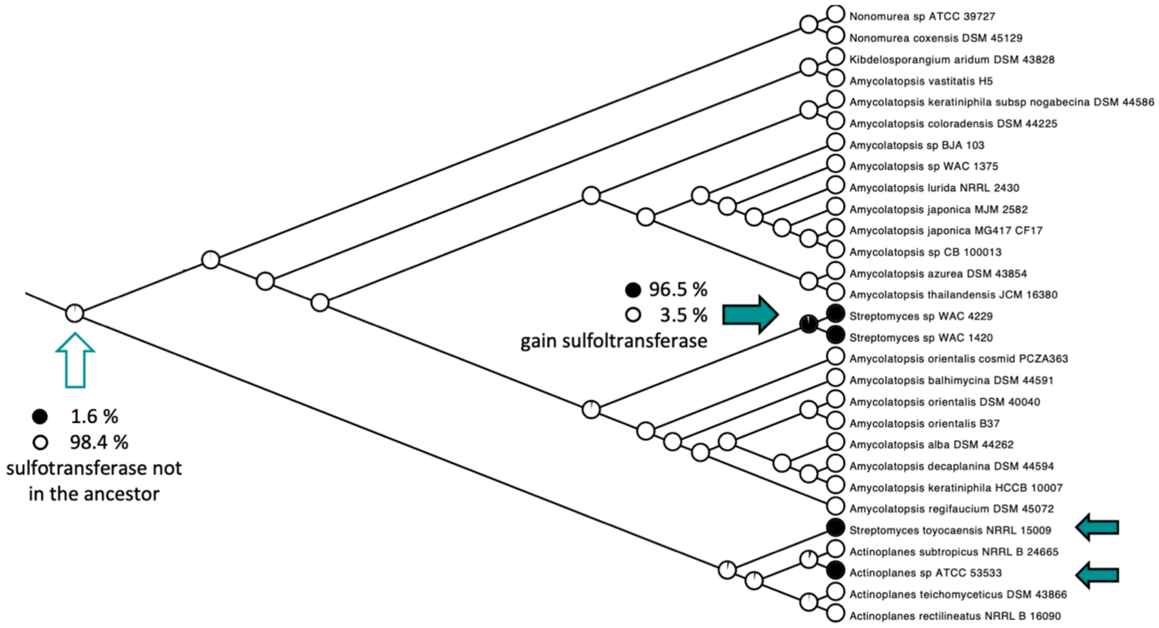

Figure S26: Ancestral state reconstruction for the sulfotransferases. Phylogenetic traits: (○) white = absent; (●) black – present.

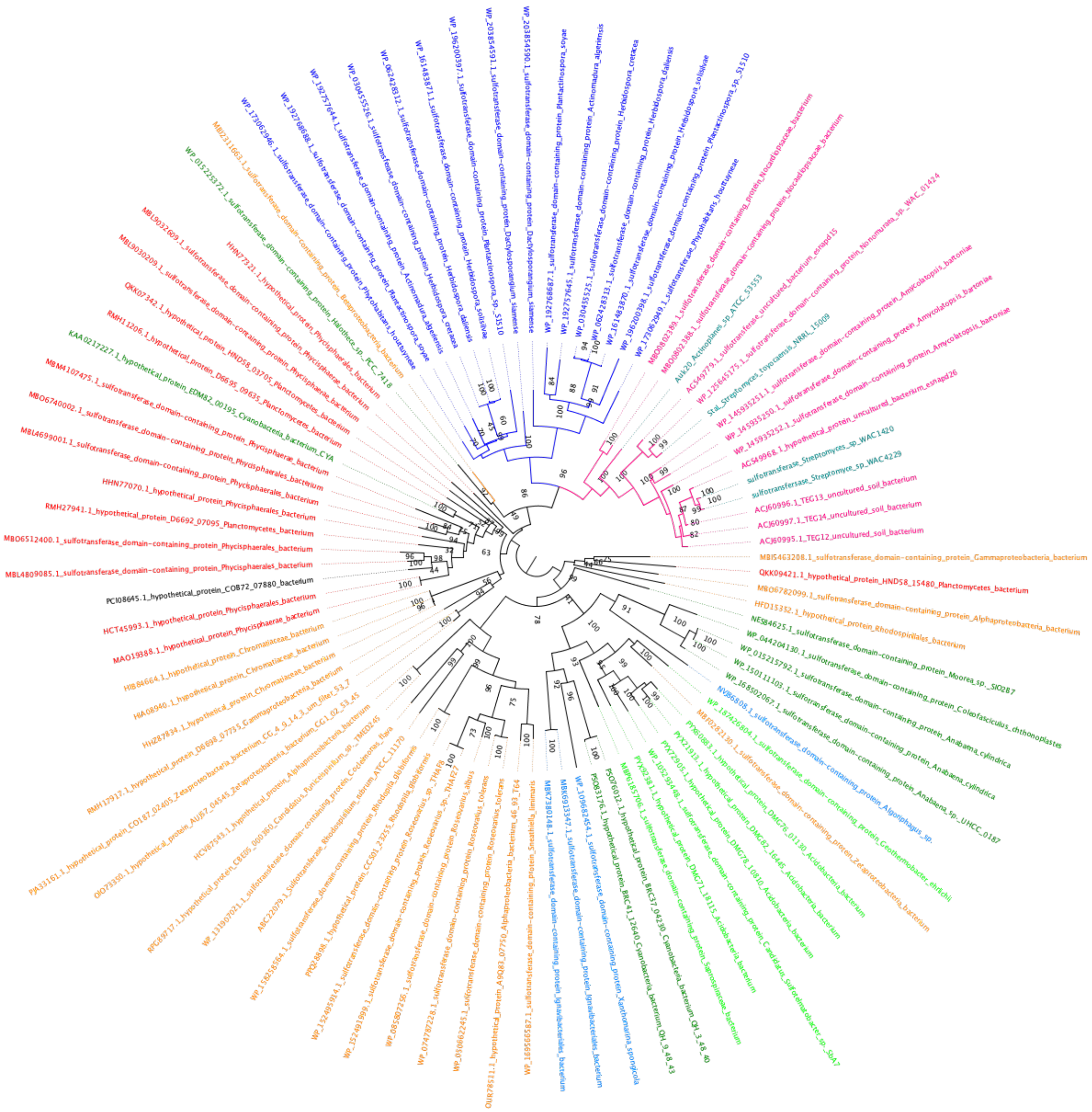

Figure S27: Maximum likelihood tree of sulfotransferases. Tree was midpoint rooted. (Alignment: Mafft e-ins-I, Blosum 62, Tree: RaxML) coloring: cyan = GPAs from this dataset; pink = other GPAs; blue = Actinobacteria; orange = Proteobacteria; red = Planctomycetes; green = Cyanobacteria; light blue = Bacteroidetes; light green = Acidobacteria.

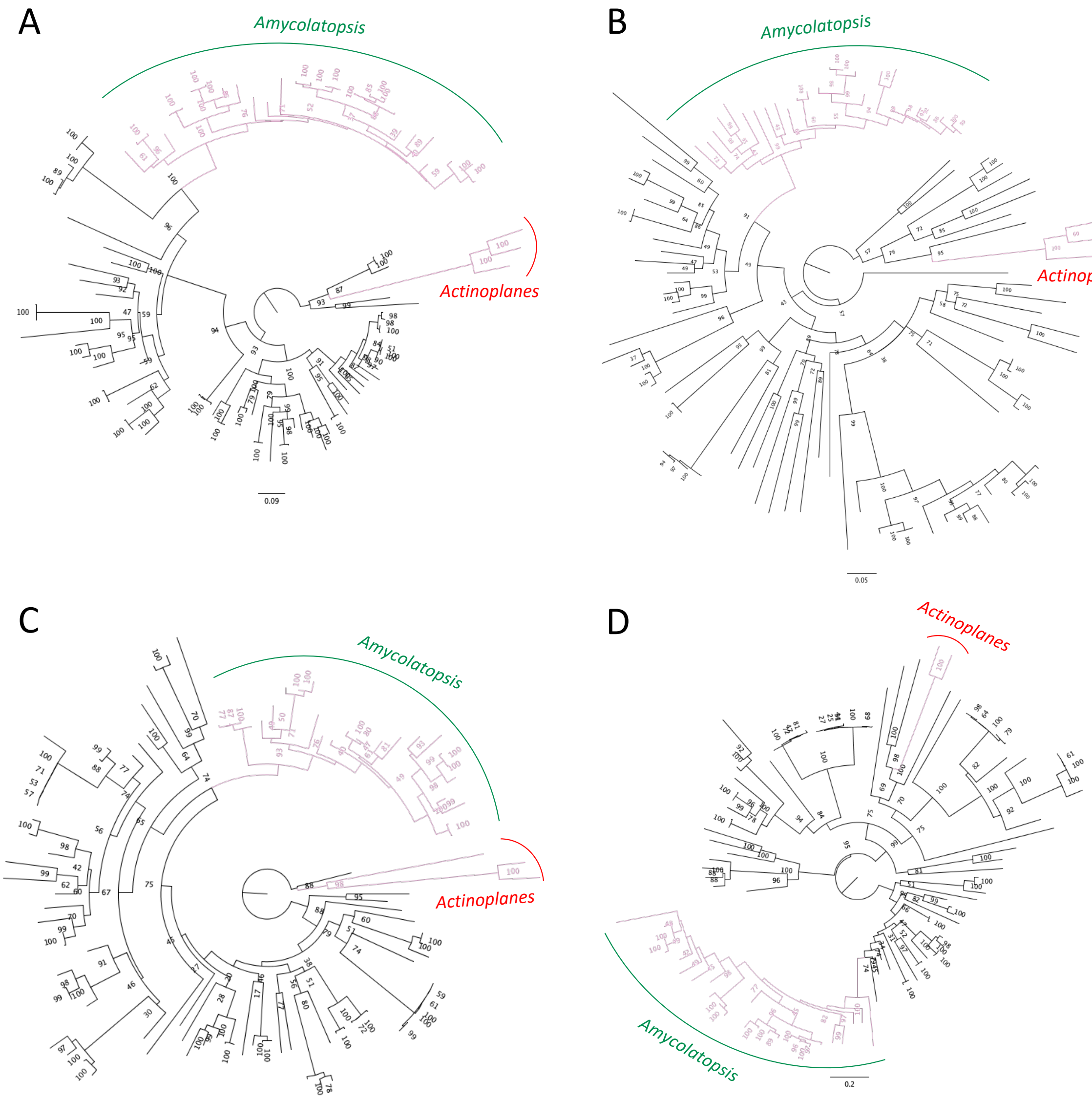

Figure S28: Maximum likelihood tree of vancosamine biosynthesis genes and homologs as determined by blast analysis A) evaA, B) evaB, C) evaD, D) evaE. Red = *Actinoplanes*; green = *Amycolatopsis*, other Actinobacteria, mostly *Streptomyces*.Trees were midpoint rooted. Sequences were aligned using the MAFFT E-INS-i algorithm (default parameters). Phylogenetic tree was calculated with IQtree, using the JTT+F+I+G4 model.

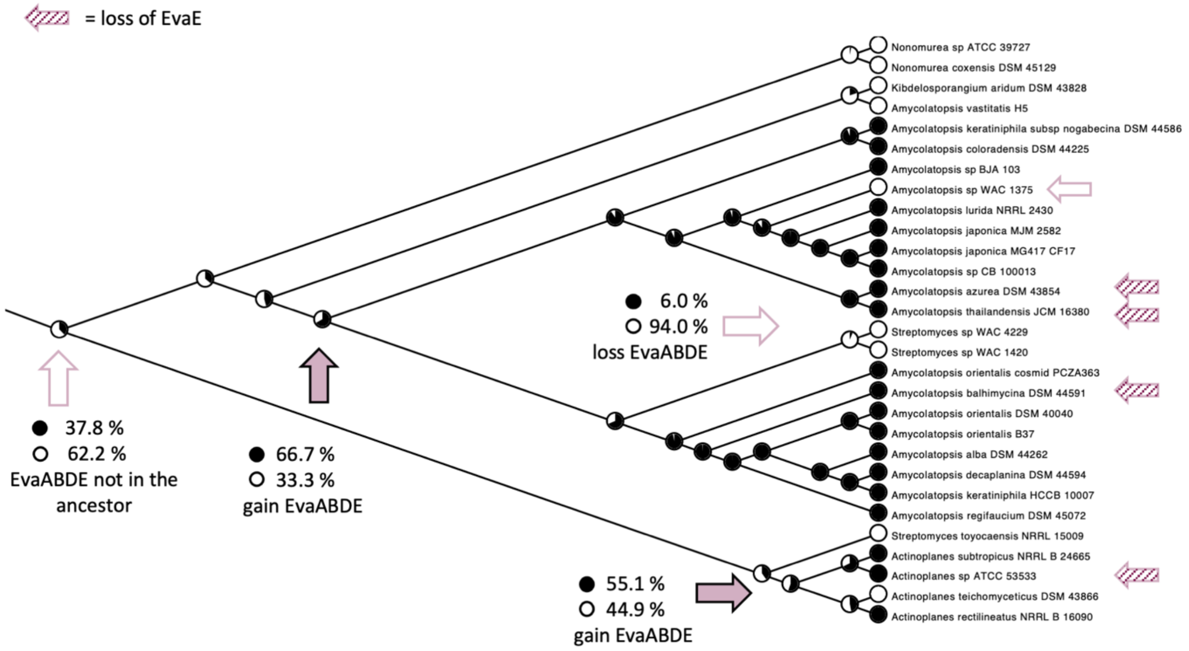

Figure S29: Ancestral state reconstruction for the vancosamine biosynthesis genes *evaABDE*. Phylogenetic traits: (○) white = absent; (●) black – present.

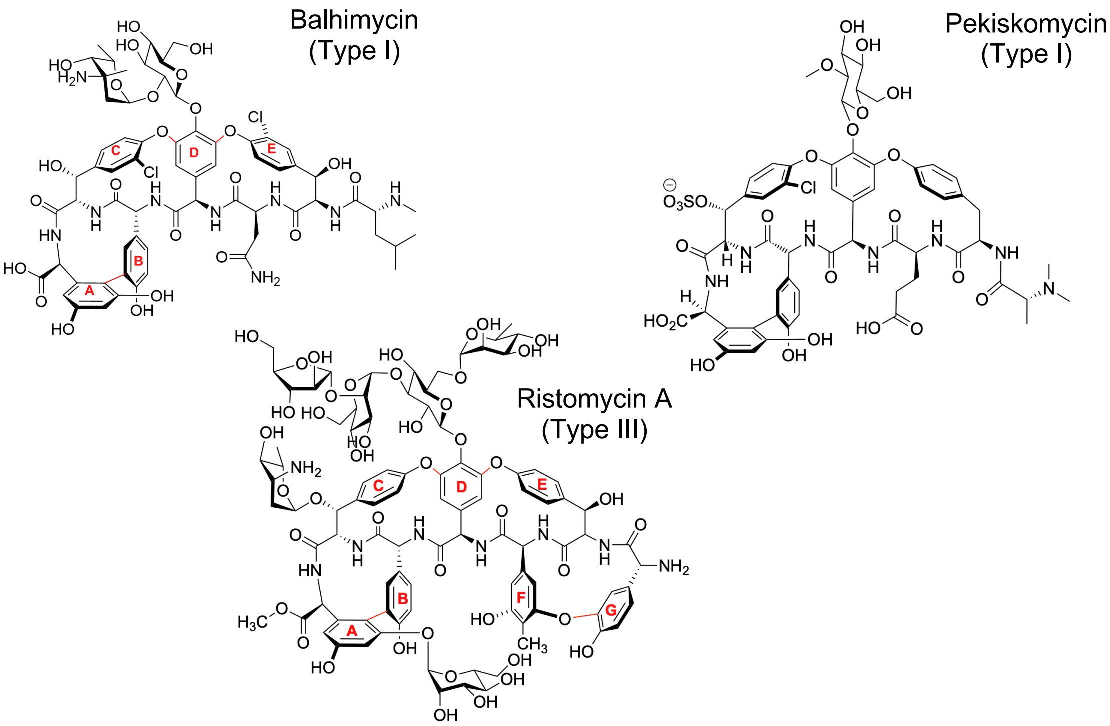
Balhimycin
(Type I)
Pekiskomycin
(Type I)
Ristomycin A
(Type III)

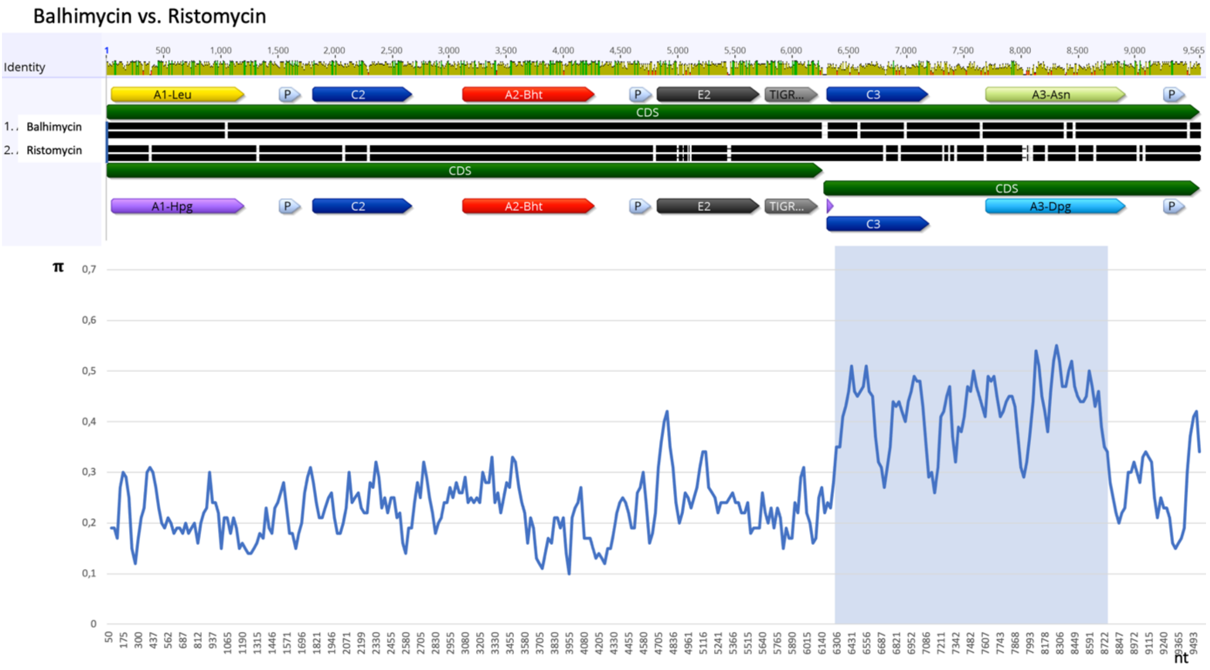
Balhimycin vs. Ristomycin
Identity
1. Balhimycin
2. Ristomycin
A1-Leu
P
C2
A2-Bht
E2
TIGR...
C3
A3-Asn
CDS
A1-Hpg
A3-Dpg
π
nt

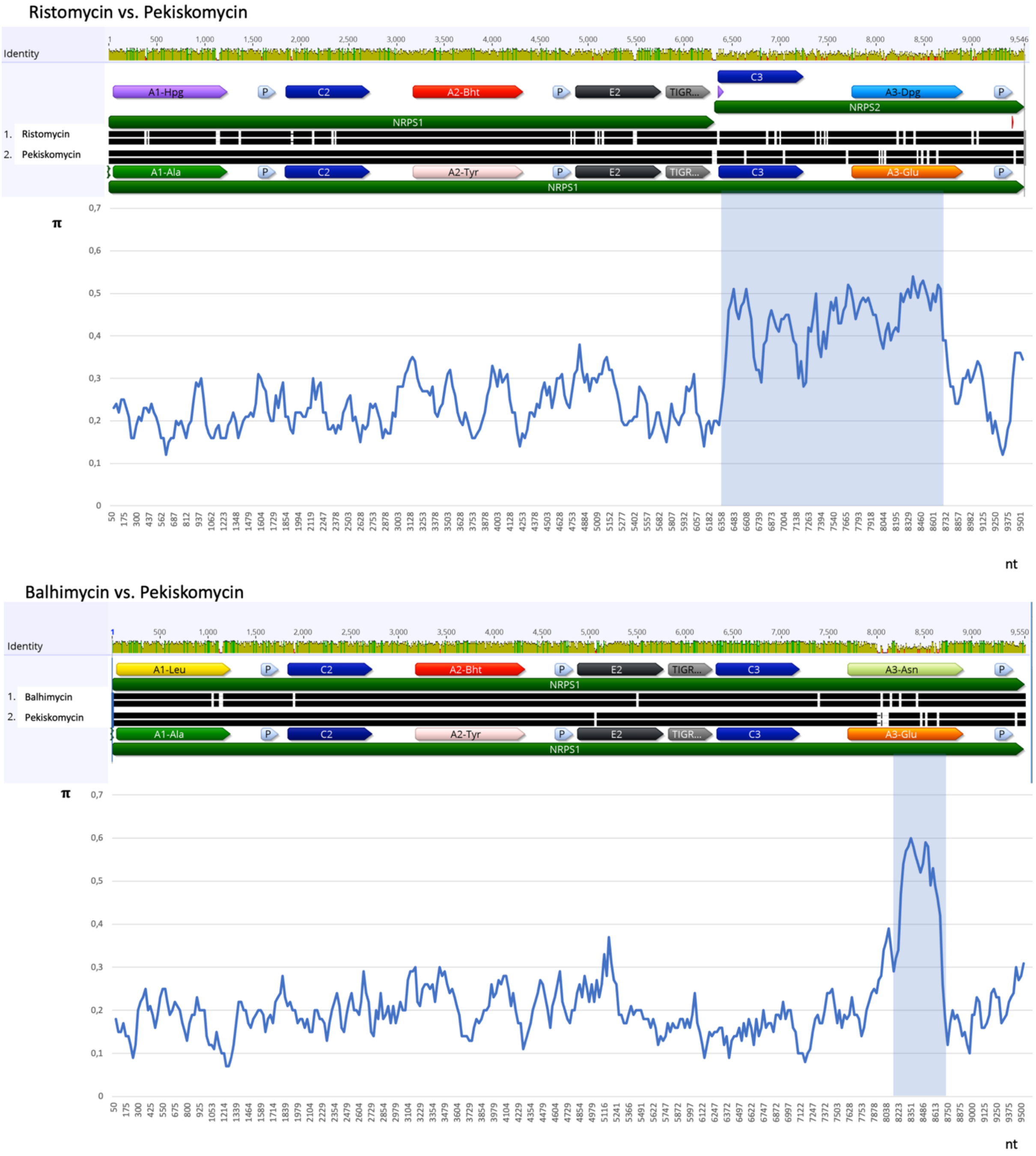

Figure S30: Sliding window analysis for identification of recombination events within modules 1-3 of the NRPS calculated with a window size of 100 nt and a step size of 25 nt. A Pi value (average number of nucleotide differences per site between two sequences) above 0.5 was considered as indication for recombination.

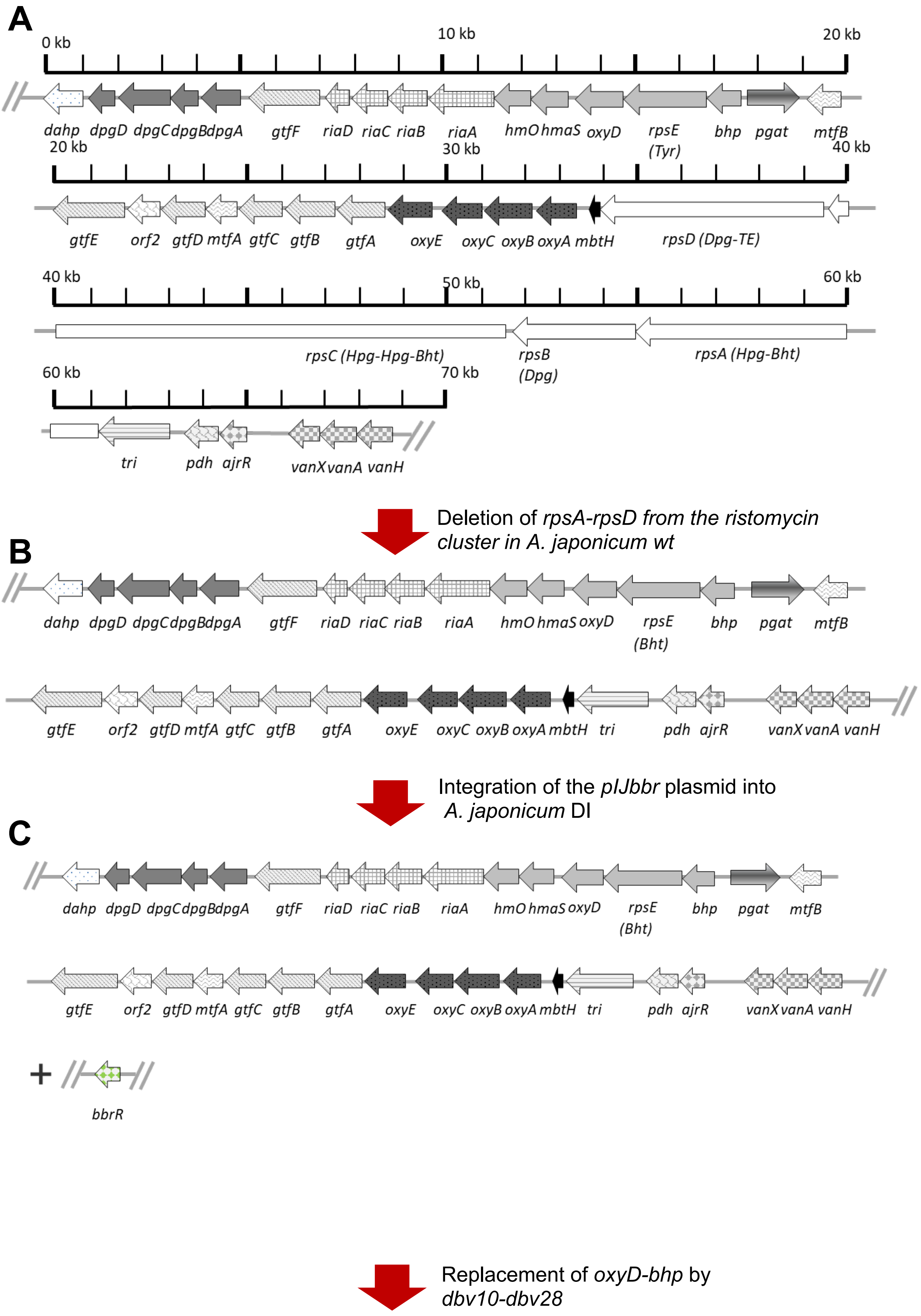

A
0 kb
10 kb
20 kb
dahp dpgD dpgC dpgB dpgA gtfF riaD riaC riaB riaA hmO hmaS oxyD rpsE (Tyr) bhp pgat mtfB
20 kb
30 kb
40 kb
gtfE orf2 gtfD mtfA gtfC gtfB gtfA oxyE oxyC oxyB oxyA mbtH rpsD (Dpg-TE)
40 kb
50 kb
60 kb
rpsC (Hpg-Hpg-Bht) rpsB (Dpg) rpsA (Hpg-Bht)
60 kb
70 kb
tri pdh ajrR vanX vanA vanH
Deletion of rpsA-rpsD from the ristomycin cluster in A. japonicum wt
B
dahp dpgD dpgC dpgB dpgA gtfF riaD riaC riaB riaA hmO hmaS oxyD rpsE (Bht) bhp pgat mtfB
gtfE orf2 gtfD mtfA gtfC gtfB gtfA oxyE oxyC oxyB oxyA mbtH tri pdh ajrR vanX vanA vanH
Integration of the pIJbbr plasmid into A. japonicum DI
C
dahp dpgD dpgC dpgB dpgA gtfF riaD riaC riaB riaA hmO hmaS oxyD rpsE (Bht) bhp pgat mtfB
gtfE orf2 gtfD mtfA gtfC gtfB gtfA oxyE oxyC oxyB oxyA mbtH tri pdh ajrR vanX vanA vanH
+
bbrR
Replacement of oxyD-bhp by dbv10-dbv28

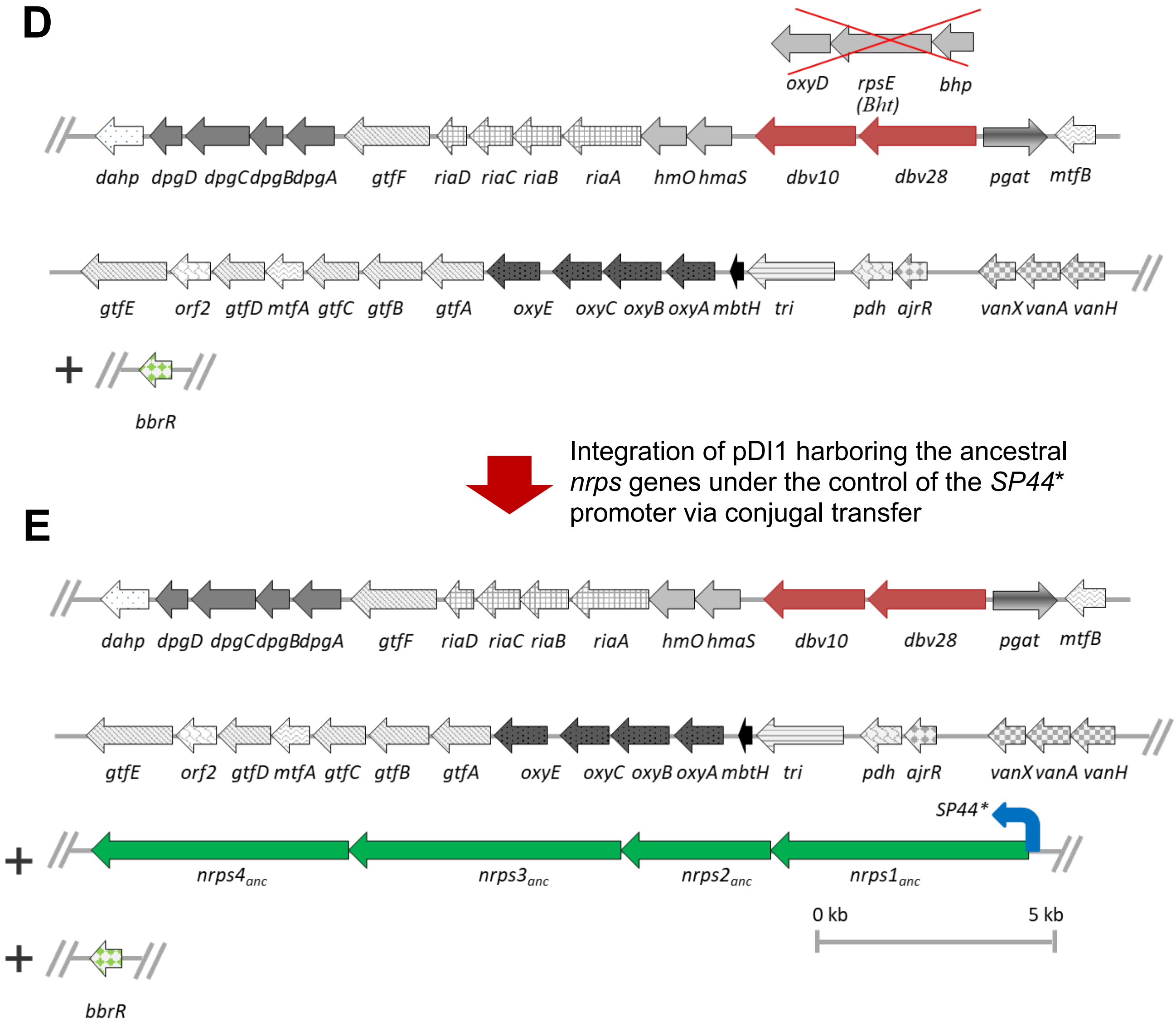

Figure S31. Stepwise modification of *A. japonicum,* the ristomycin producer strain, to enable the heterologous production of paleomycin-ristomycin hybrids (**A-E**). (A) Schematic representation of the ristomycin cluster encoded in the genome of *A. japonicum*. (B) Construction of a ristomycin deficient mutant by the deletion of the native ristomycin biosynthetic nrps genes. (C) Integration of a copy of the pathway specific regulator bbr cloned on the pIJ plasmid, into the chromosome of A. japonicum. (D) Replacement of the oxyD, rpsE and bhp genes by dbv10-dbv28 from *N. gerenzanensis* through homologous recombination using the pGUS system. (E) Integration of pDI1 plasmid harbouring the ancestral nrps genes under the control of the SP44* promoter into the genome of the modified *A. japonicum* via intergeneric conjugation, resulting in paleomycin production.

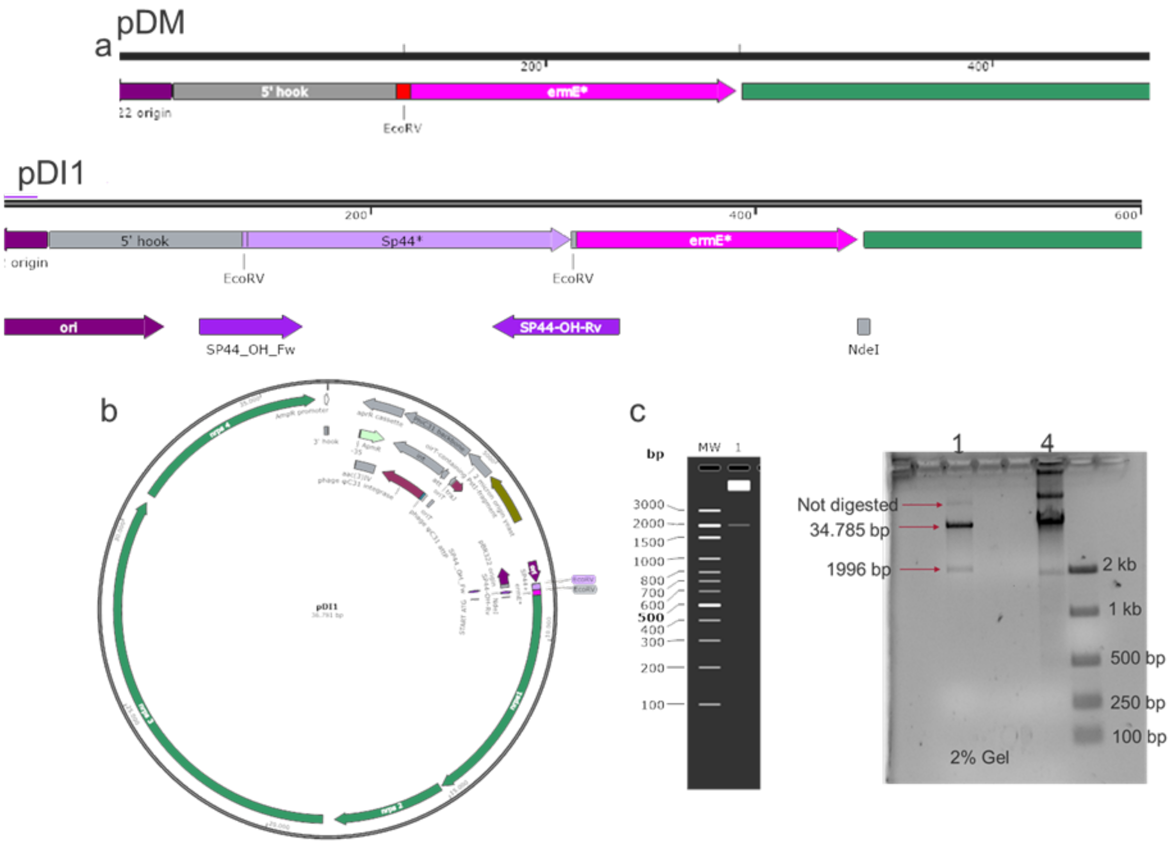

Figure S32. Integration of the Sp44* promoter into *p3SV_nrps*$_{anc}$ *(pDM)* resulting in *pDI1.* a) Schematic representation of the in-fusion cloning mediated insertion of *Sp44** promoter into pDM, downstream of *ermE*p,* shown in violet. The pDM plasmid was linearized by *EcoRV* and the *Sp44**. b) Plasmid map of *pDI1* after insertion of *Sp44** promoter. c) Agarose gel of *pDI1* plasmid extracted from *E. coli HST08,* line 1 and 4 show the expected fragment size of 1.9 kb and 34.7 kb after digestion with *NdeI.*

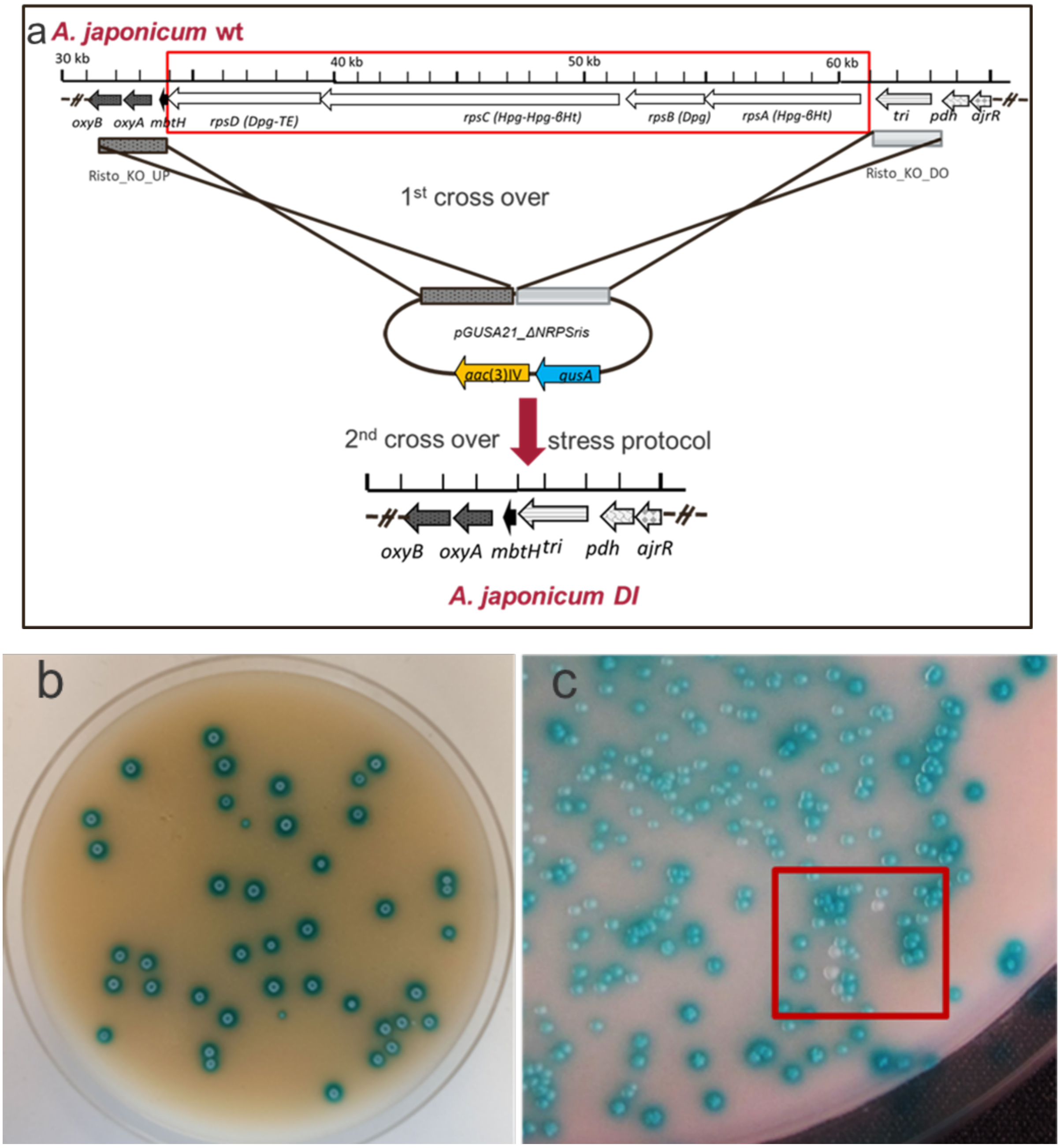

Figure S33. Steps involved in deleting the rpsA-rpsD genes. a) Scheme of the *rpsA-rpsD* deletion strategy. 1.3 and 1.5 kb upstream and downstream fragments of *rpsD* and *rpsA* NRPS-genes were cloned into the pGUSA21 vector. The 1st crossover is due to homologous recombination with one of the cloned fragments. The 2nd crossover leads to disintegration of the plasmid and eventually to the deletion of *nrps*-genes. It can be provoked by stress effects on the cells to (e. g. formation of protoplasts, cultivation at 37°C. b) Selection of mutants with plasmids integrated into the chromosome is performed on plates containing apramycin and X-gluc (5-bromo-4-chloro-1H-indol-3-yl β-D glucopyranosiduronic acid, (blue colonies); c) The occurrence of the 2nd crossover (nrps$_{ris}$ deletion) is confirmed by X-gluc selection (white colonies) on MS-agar plates (Ø10 cm) overlaid with 20 mM X-gluc.

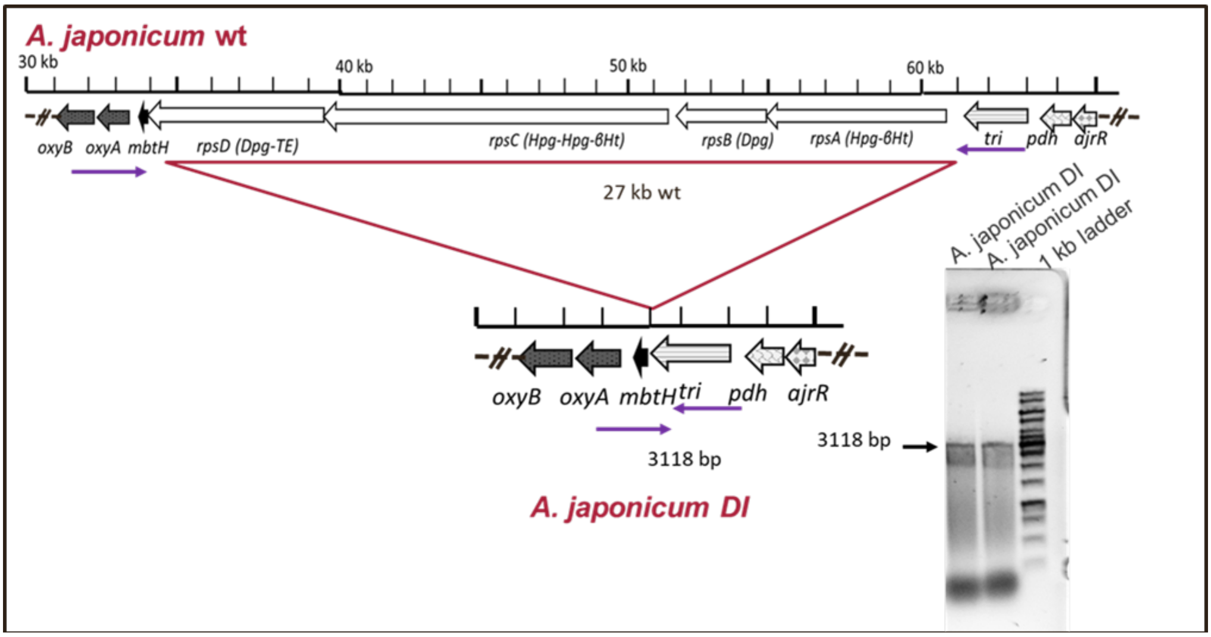

Figure S34. Analysis for verification of the in-frame deletion of NRPS genes from ristomycin cluster. The deletion of the NRPS genes from the ristomycin cluster was confirmed by PCR using the primer pair *A. jap*Δ-CytP450 Fw/*A. jap*Δ-ABC-Rv (**Table S3**). A 3118 kb fragment was amplified using genomic DNA of *A. japonicum DI* (line 1 and 2); 1 kb ladder line 3.

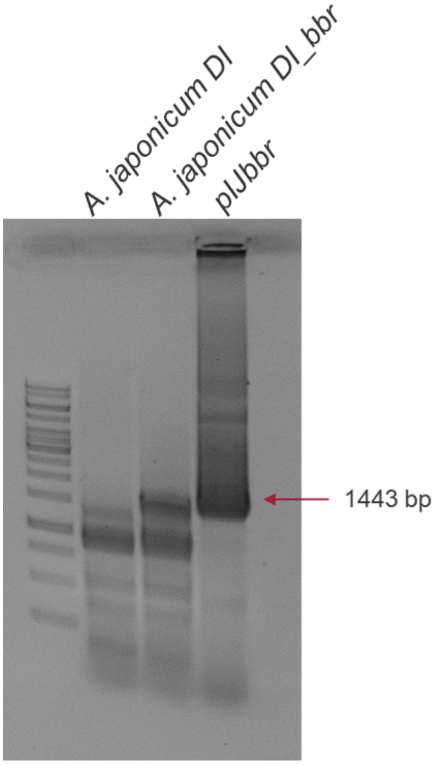

Figure S35. Integration of pIJ*bbr* into *A. japonicum DI.* Agarose gel of the PCR confirming the integration of the *pIJbbr* plasmid into *A. japonicum DI*, 1 kb ladder line 1, no amplification for *A. japonicum DI*, line 2, 1443 bp fragment amplified in *A. japonicum DI_pIJbbr*, line 3 and 1443 bp fragment amplified in the positive control *pIJbbr* line 4.

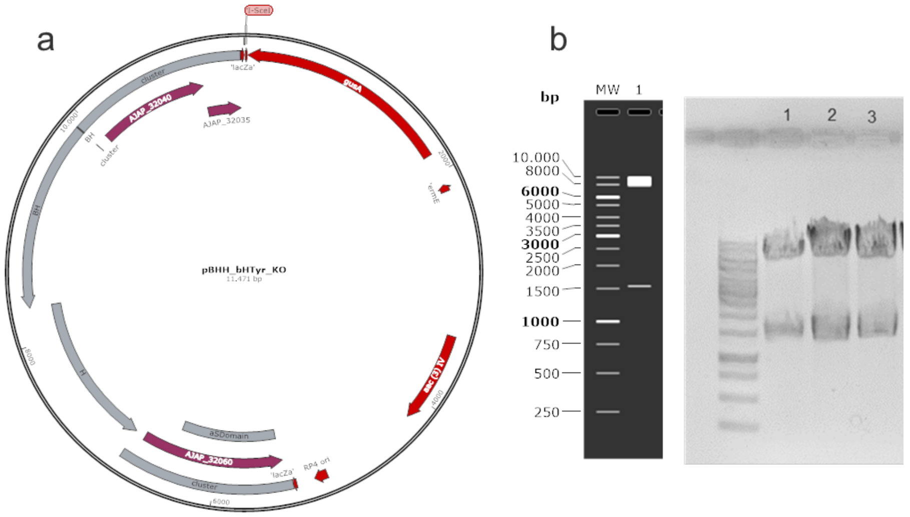

Figure S36. Construction and verification of the pBHH_bHTyr_KO vector**.** a) Plasmid map of pBHH_bHTyr_KO designed to delete the *oxyD, rpsE and bhp* genes from the ristomycin cluster and to replace by the halogenase and β-hydroxylase genes (BN4615_P2170 and BN4615_P2189) via homologous recombination. The primers Aj-32060 fw, Aj-32060 rv, Aj-32040 fw and Aj-32040 rv were used to amplify the 1.53 und 1.56 kb flanking regions for the homologous recombination and the primers BHH-fw, BHH-rv for the amplification of the fragment containing both genes, the halogenase and the β-hydroxylase. The pGUSA21 plasmid was linearised by *HindIII* and *NdeI* and used for in-fusion with the amplified fragments. b) Agarose gel of pBHH_bHTyr_KO plasmid extracted from *E. coli HST08,* line 1 to 3 show the expected fragment size of 9.9 kb and 1.5 kb after digestion with *NdeI.*

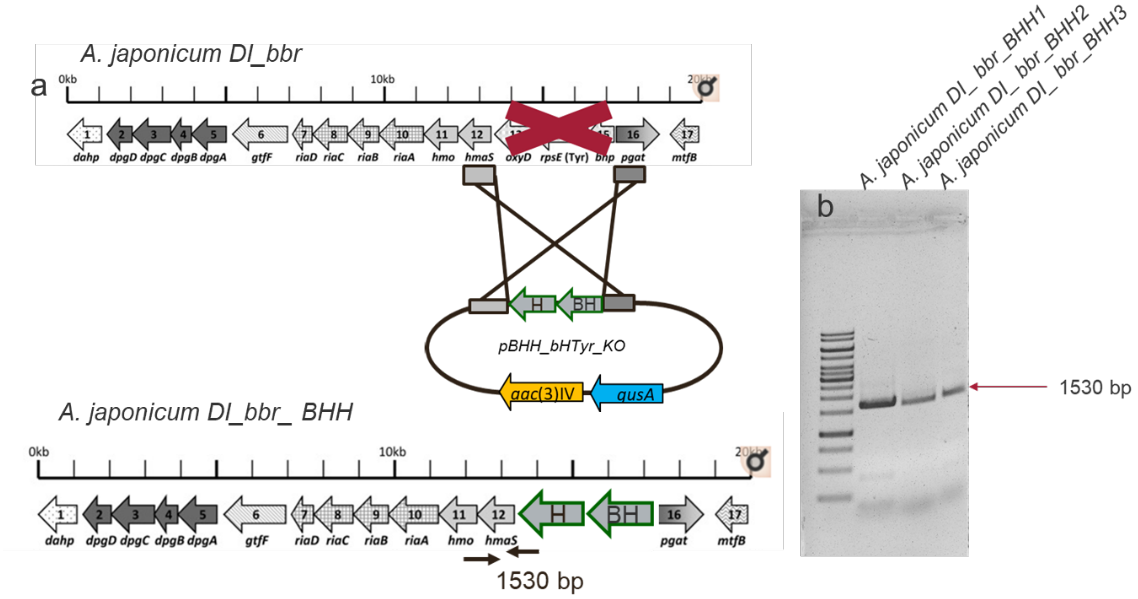

Figure S37. Construction of *A. japonicum DI_* bbr_BHH**.** a) Schematic representation of deletion of the *oxyD, rpsE and bhp* genes in *A. japonicum DI_*bbr and replacement by the halogenase and β-hydroxylase genes (BN4615_P2170 and BN4615_P2189) via homologous recombination**.** b) Agarose gel of the PCR confirming the integration of the halogenase and β-hydroxylase genes into *A. japonicum DI_*bbr, 1 kb ladder line 1, 1530 bp fragment amplified in *A. japonicum DI_bbr_BHH* for different transconjugants, line 2-4.

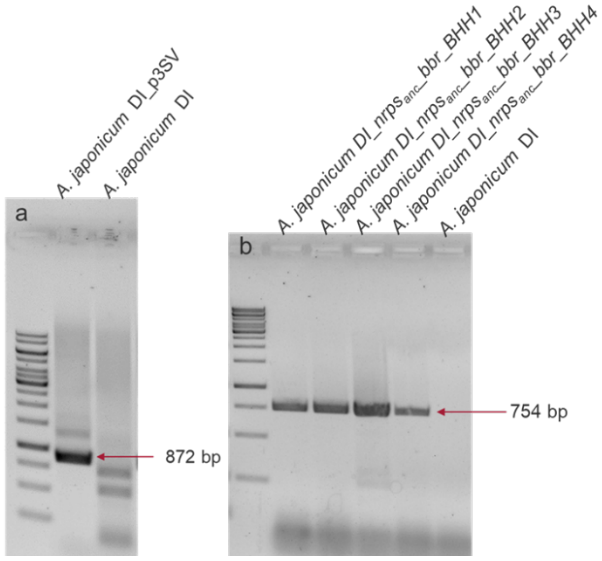

Figure S38. Analysis of the integration of p3SV and pDI1 into *A. japonicum* DI**.** a) Agarose gel of the PCR confirming the integration of p3SV plasmid into *A. japonicum* DI*,* 1 kb ladder line 1, 872 bp fragment amplified in p3SV, line 2 and no amplification for the negative control line 3; b) Agarose gel of the PCR confirming the integration of pDI1 plasmid into *A. japonicum* DI_nrps$_{anc}$*_bbr_BHH*, 1 kb ladder line 1, 754 bp fragment amplified in pDI1, line 2-5 for different transconjugants and no amplification for the negative control, line 6.

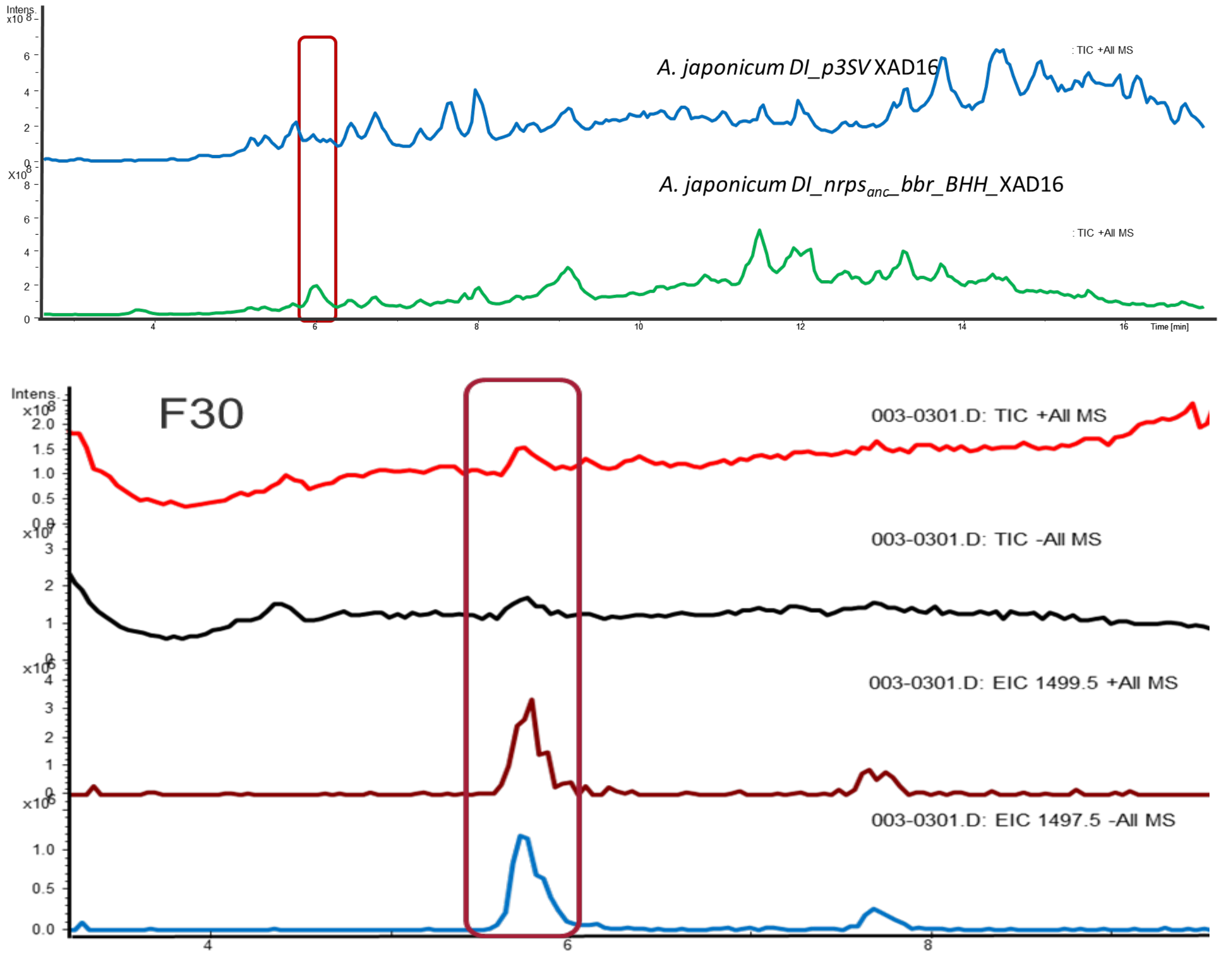

Figure S39. Total ion chromatograms of *A. japonicum DI_p3SV* and *A. japonicum DI_nrps$_{anc}$_bbr_BHH* (upper) and total plus extracted ion chromatograms of F30 from *A. japonicum DI_nrps$_{anc}$_bbr_BHH* after LH20 (lower); marked peak show GPA elution at tR=5.6-6.0 min. Relative intensity is shown on the y axes.

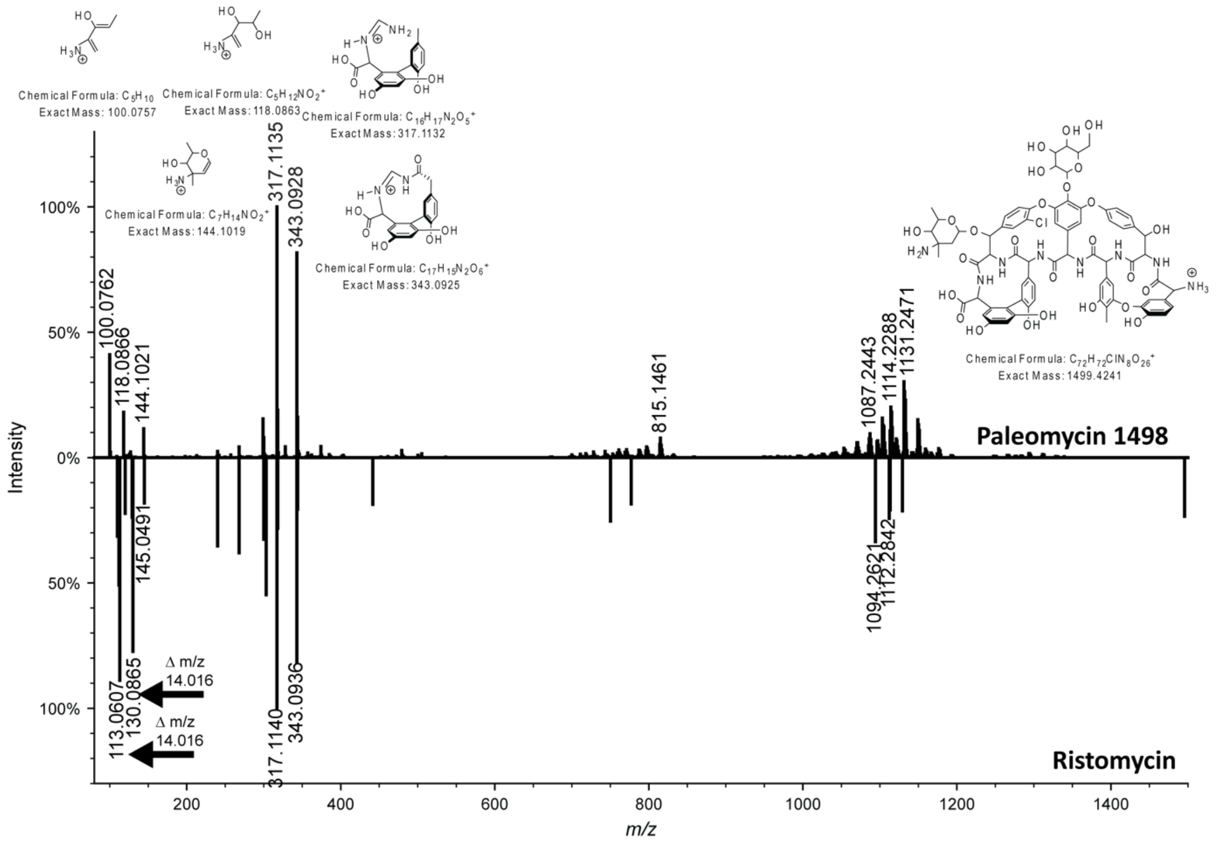

Figure S40. Comparative MS^2 fragmentation of the ristomycin/paleomycin hybrid GPA (upper) and ristomycin A that is naturally biosynthesised by *A. japonicum* (lower). Key lower MW fragments are indicated on the left-hand side of the figure.

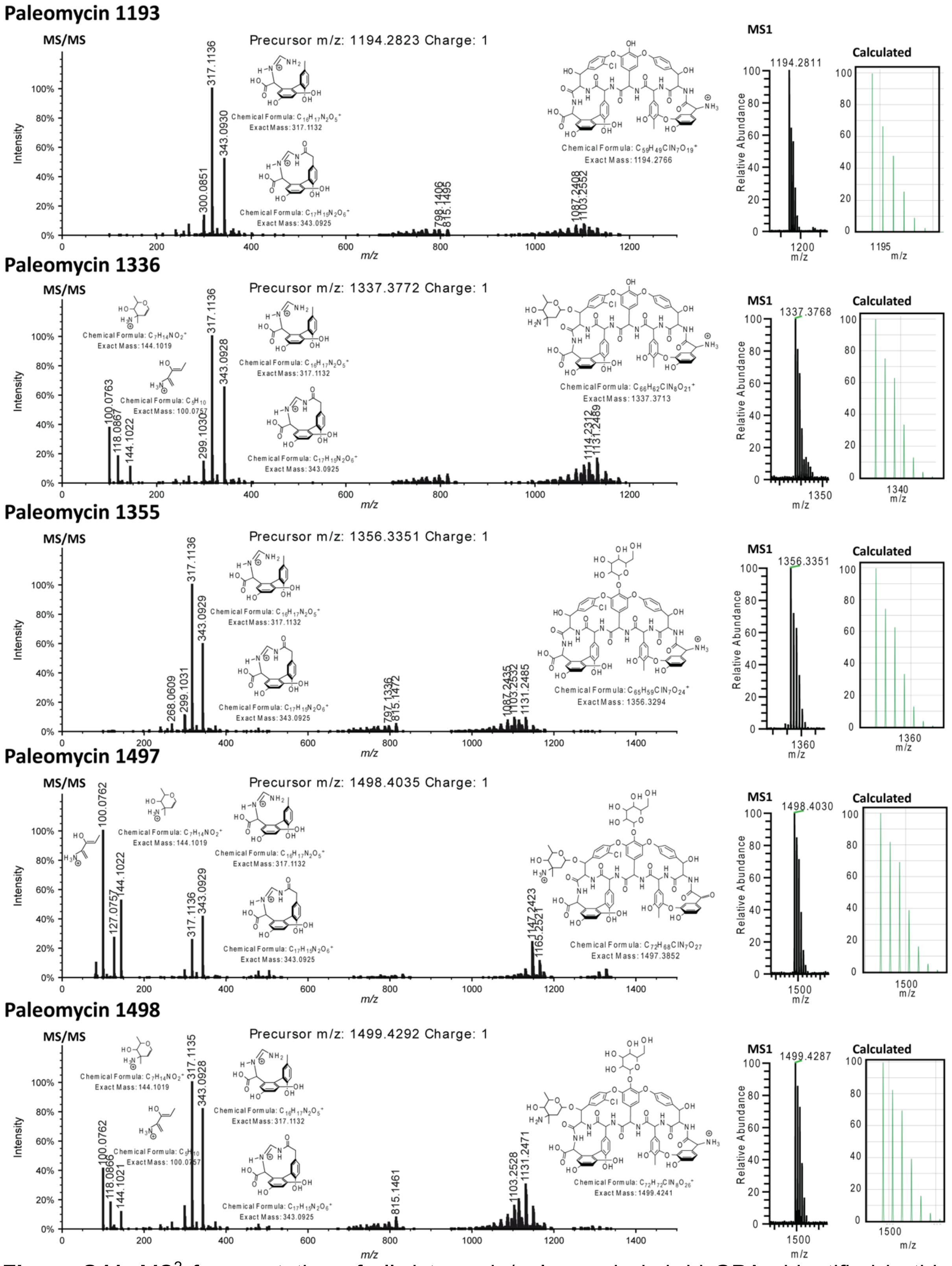

Figure S41. MS[2] fragmentation of all ristomycin/paleomycin hybrid GPAs identified in this work and indicated in the network diagram shown in **Figure 5**. Major differences include the presence of up to two different glycosyl units and deamination at the peptide N-terminus. A single methylation is detected, with fragmentation analysis suggesting methylation at Dpg_3.

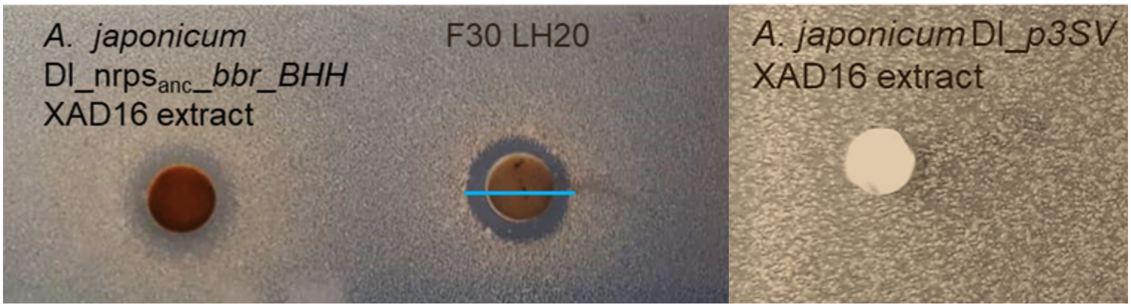

Figure S42. Representative plate of agar diffusion assay against *B. subtilis* ATCC6633 as test organism. The assay was performed with XAD16 extract of the *A. japonicum* DI_nrpsanc_bbr_BHH and fraction 30 (F30) eluate from the LH20 column containing the ancestral GPA and with *A. japonicum* DI_p3SV as negative control.

A) A-domains

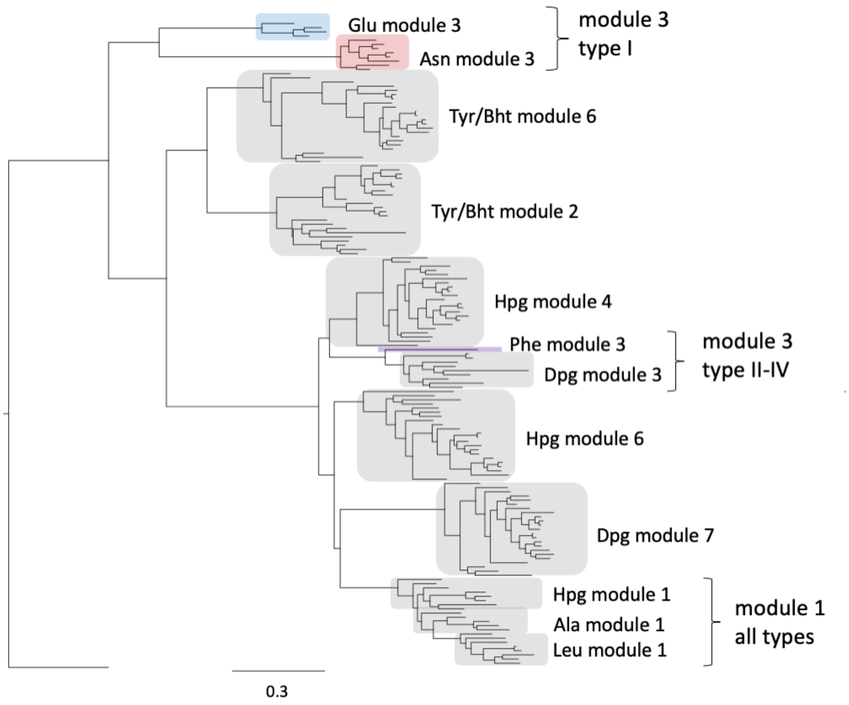

B) C-domains

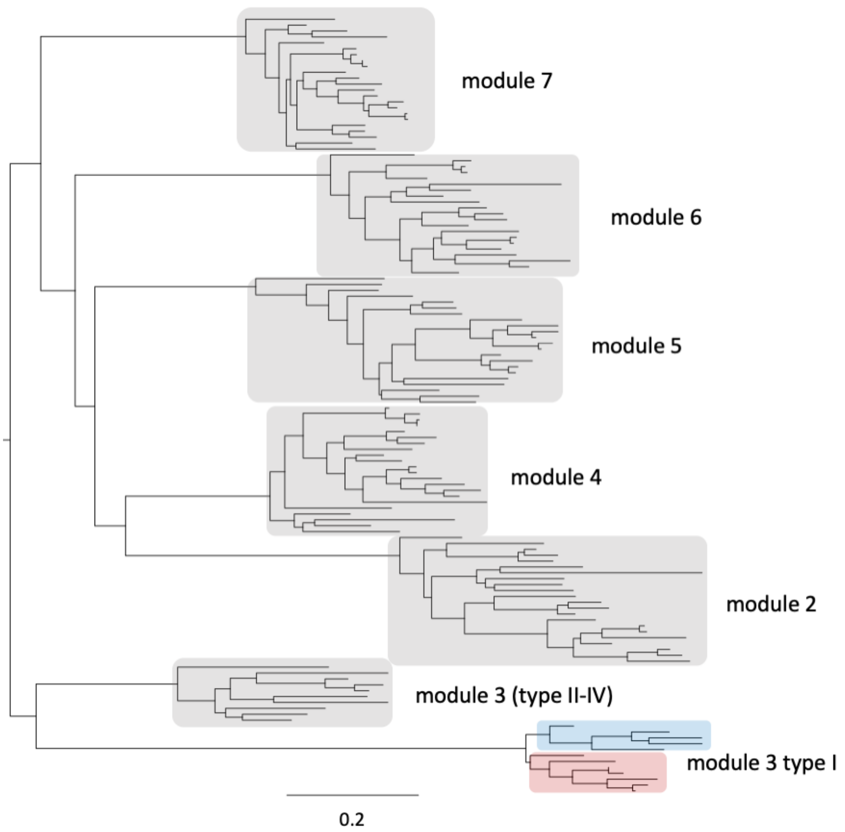

Figure S43. Phylogeny of GPA A- and C-domains. Maximum likelihood trees of A) GPA A-domains and B) GPA C-domains. Module 3 is highlighted in red (vancomycin-type I), blue (pekiskomycin-type I) or purple (nogabecin-type II). Sequences were aligned using the MAFFT E-INS-I algorithm (default parameters). Phylogenetic trees were calculated with IQtree using the JTT+F+I+G4 model.

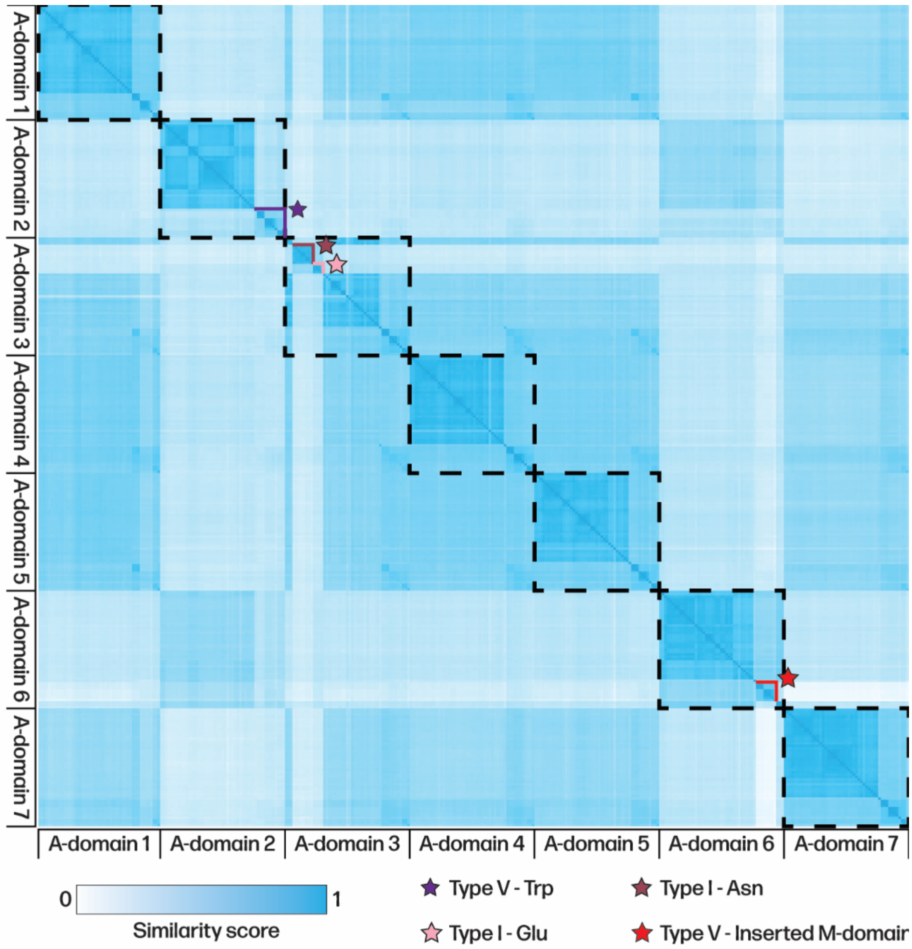

Figure S44. Matrix comparing similarity using a normalised similarity score. The similarity score between each sequence was generated using SIAS tool (http://imed.med.ucm.es/Tools/sias.html). All A-domains used in generating the phylogenetic tree shown in **SI Figure S45.**

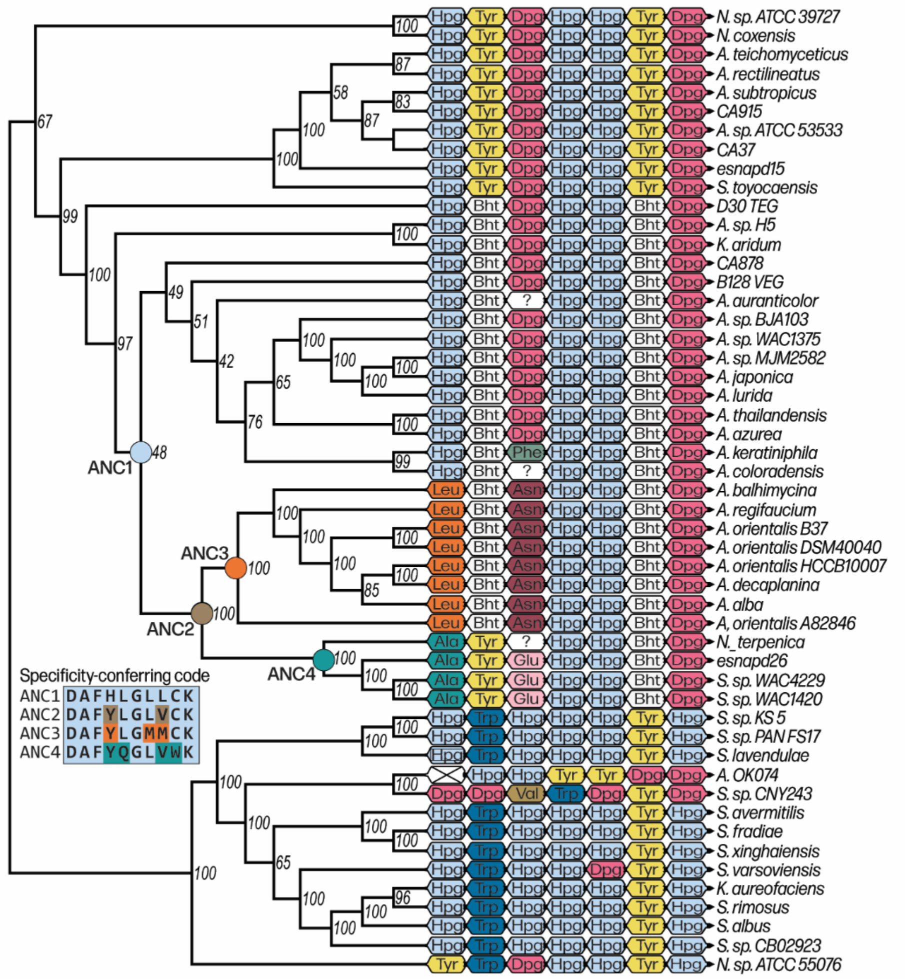

Figure S45. Full phylogeny of NRPSs producing a GPA scaffold. Each branch is annotated with the predicted heptapeptide scaffold purely based on the A-domain specificity conferring code. Tree was constructed by means of a partitioned tree search using IQ-tree v1.6.3 [12,13]. Using both protein and codon sequences, a substitution model was chosen for constructing the phylogeny. IQ-tree has an in-build model finder that was used to automatically pick the best substitution models [14]. Bootstrapping was applied to measure the robustness of the tree, using 500 bootstrap repetitions [15]. The tree was prepared with dendroscope [10].

Average of reconstruction = 0.91

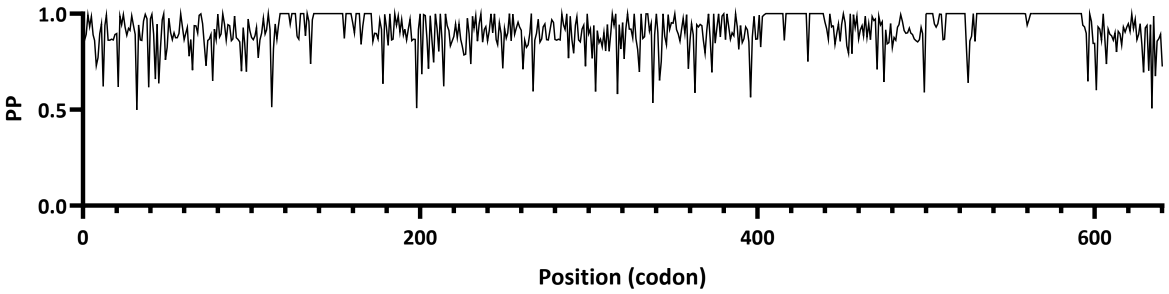

>ANC1
VLDLFARQVDRTPDAVAVVDGDRVLTYRQLDELAGRLSGRLIGRGVRRGDRVAVMMDRSA
DLLVALLAVWKAGAAYVPVDAAYPARRVAFMVADSGASLMVCSAATRDGVPEGIESIVVTD
EGACDASAVTVRPGDLAYVMYTSGSTGTPKGVAVPHRSVAELVGNPGWAVEPGDAVLMH
APHAFDASLFEIWVPLVSGARVVIAEPGPVDARRLREAVAAGVTRAHLTAGSFRAVAEESP
ESFAGLREVLTGGDLVPAHAVERVREACPRVRIRHLYGPTETTLCATWHLLEPGDVMGPVL
PIGRPLPGRRAHVLDASLRPVAPGVVGDLYLSGAGLADGYLDRAGLTAERFVADPSAPGKR
MYRTGDLAQWTADGELLFAGRADDQVKIRGFRIEPGEIEAALTAQPDVHEAVVVAIDGRLIG
YVVADGDADPVLIRERLGAVLPEYMVPAAVITLDALPLTGNGKVDRAALPAPDFAANAT

Average of reconstruction = 0.90

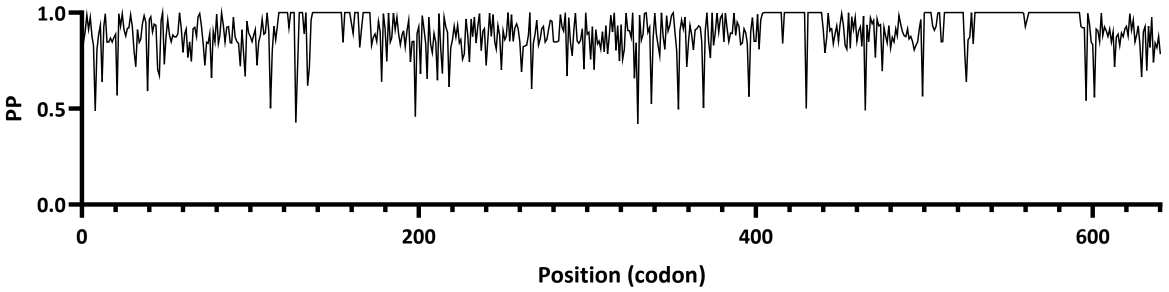

>ANC2
VLDLFARHVDRTPDAVAVVDGDRVLTYRQLDELAGRLSGRLISRGVRRGDRVAVMMDRSA
DLLVALLAVWKAGAAYVPVDAAYPARRVAFMVADSGASLMVCSAATRDGVPEGIESIVVTD
EGACDASAVTVRPGDLAYVMYTSGSTGTPKGVAVPHRSVAELVGNPGWAMEPGEAVLMH
APHAFDASLFEIWVPLVSGARVVIAEPGPVDARRLREAVAAGVTRAYLTAGSFRAVAEESP
ESFAGLREVLTGGDLVPAHAVERVREACPRARIRHLYGPTEATVCATWHLLEPGDVMGPV
LPIGRPLSGRRAHVLDESLRPVGPGVVGDLYLSGAGLADGYLNRAGLTAERFVADPSAPGK
RMYRTGDLAQWTADGELLFAGRADHQVKIRGFRIEPGEIEAALTAQPDVHDAVVVAIDGRLI
GYVVADGDADPVLIRERLGAVLPEYMVPAAVIALDALPLTGNGKVDRAALPAPEFAANAT

Average of reconstruction = 0.88

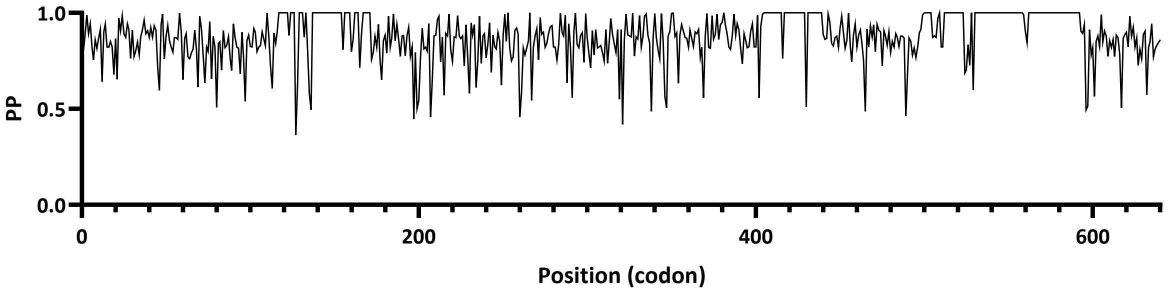

>ANC3
VLDLFARHVDRTPDAVAVADGDRVLTYRQLDELAGRLSGRLISRGVRRGDRVAVMMDRSA
DLVVALLAVWKAGAAYVPVDAAYPAPRVAFMVADSAASLMVCSAATRDGVPEGIESIVVTD

EDACDASAATVRPGDLAYVMYTSGSTGTPKGVAISHGSVAELVGDPGWAMEPGEAVLMH
SPHAFDASLFEIWTPLASGARVVIAEPGSVDARRLREAAAAGVTRVYLTAGSFRAVAEESPE
SFAAFREVLTGGDVVPAHAVERVREACPRARIRHMYGPTEATMCATWHLLEPGDVMGPVL
PIGRPLAGRRVQVLDESLRPVEPGVVGDLYLSGGLAEGYFNRAGLTAERFVADPSAPGQR
MYWTGDLAQWTADGELLFAGRADHQVKIRGFRIEPGEIEAALIAQPDVHDAVVAAIDGRLIG
YVVADGDVDPVLIRERLGAVLPEYMVPAAVIALDALPLTGNGKVDRAALPAPEFAANAT

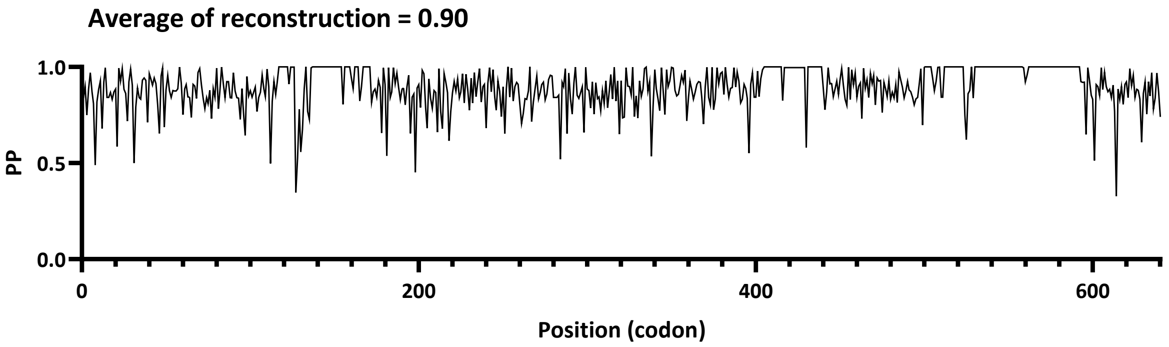

>ANC4
VLELFARHVDRTPDAVAVVDGDRVLTYRQLDELAGRLSGRLISRGVRRGDRVAVMMDRSA
DLLVALLAVWKAGAAYVPVDAAYPARRVAFMVADSGASLMVCSVATRDGVPEGIESIVVDA
ATDEGACDASAVTVRPGDLAYVMYTSGSTGTPKGVAVPHRSLAELVGNSGWAMEPGEAV
LMHAPYAFDASMFEIWVPLVWGARVVIAGPGPVDARRLREAVAAGVTRAYLTAGSFRAVA
EESPESFAGLREVQTGGDLVPAHAVERVREACPRARIRHLYGPTEATVWATWHLLEPGDV
MGPVLPIGRPLSGRRAHVLDESLRPVGPGVVGELYLSGAGLADGYLNRAGLTAERFVADP
SAPGKRMYRTGDLAQWTADGELLFAGRVDHQVKIRGFRIEPGEIEAALTAQPDVHDAVLVA
IDGRLIGYVVADGDADPVLIRERLGAVLPEYMVPAAVIALDALPLTGNGKVDRAALPAPEFAA
NAT

Figure S46. Sequences of the maximum likelihood ancestors are shown for the four characterised ancestral enzymes. Graphs display the posterior probabilities of each reconstructed codon for ANC1, ANC2, ANC3 and ANC4.

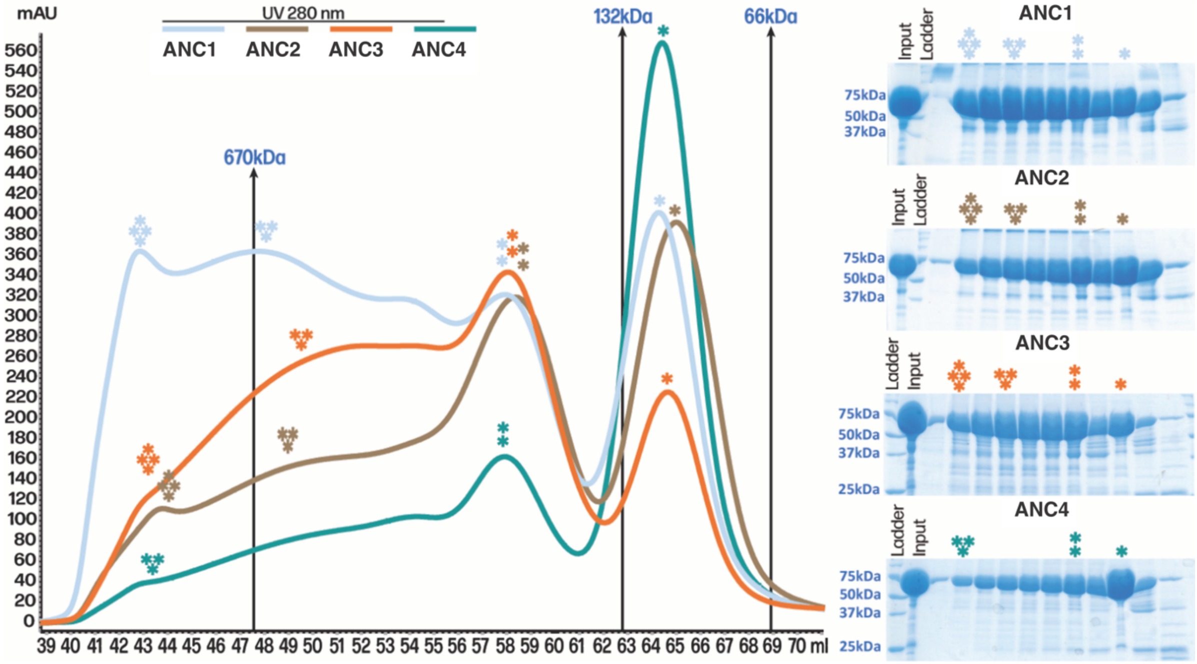

Figure S47. ANC1-4 showed polydisperse elution profiles during size exclusion. Each ancestor was expressed with an N-terminal hexahistidine-SUMO tag and a C-terminal STREP tag and co-expressed with the MbtH-like protein tcp13. The tagged ancestors have a molecular weight of approximately 72 kDa. On the left, overlay of chromatogram from size exclusion. Elution times obtained from protein standard is annotated on chromatogram (Thyroglobulin 670 kDa, BSA dimer 132 kDa and BSA 66 kDa). On the right, SDS-PAGE gels of fractions after size exclusion using an SRT-10 SEC-300 (SEPAX) column with fractions annotated with coloured asterisks denoting which peak on the chromatogram they eluted from. Input shows sample loaded on the SRT-10 SEC-300 (SEPAX) column and was obtained from affinity chromatography using a gravity flow column loaded with Ni-NTA resin.

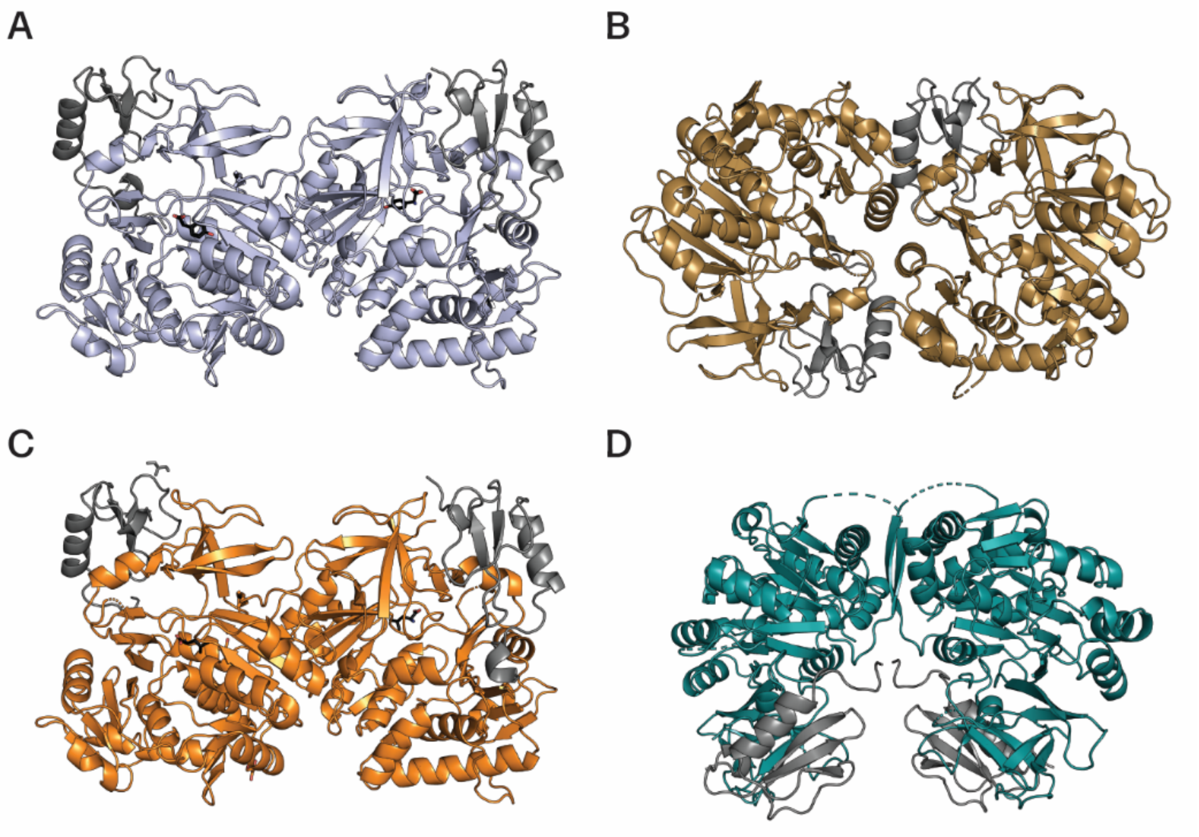

.

Figure S48. Asymmetric unit. The asymmetric unit of **A)** A1$_{core-tei}$, **B)** A1$_{core-ANC2}$, **C)** A1$_{core-ANC3}$ and **D)** ANC4$_{core}$ each contains two heterodimers of the Mbth-like protein Tcp13 and A$_{core}$. Tcp13 is shown as dark grey and A1$_{core-tei}$ shown as light blue, A1$_{core-ANC2}$ as sand, A1$_{core-ANC3}$ as orange and ANC4$_{core}$ as teal. The bound substrate is shown in black for L-Hpg and D-Leu for A1$_{core-tei}$ and A1$_{core-ANC3}$, respectively.

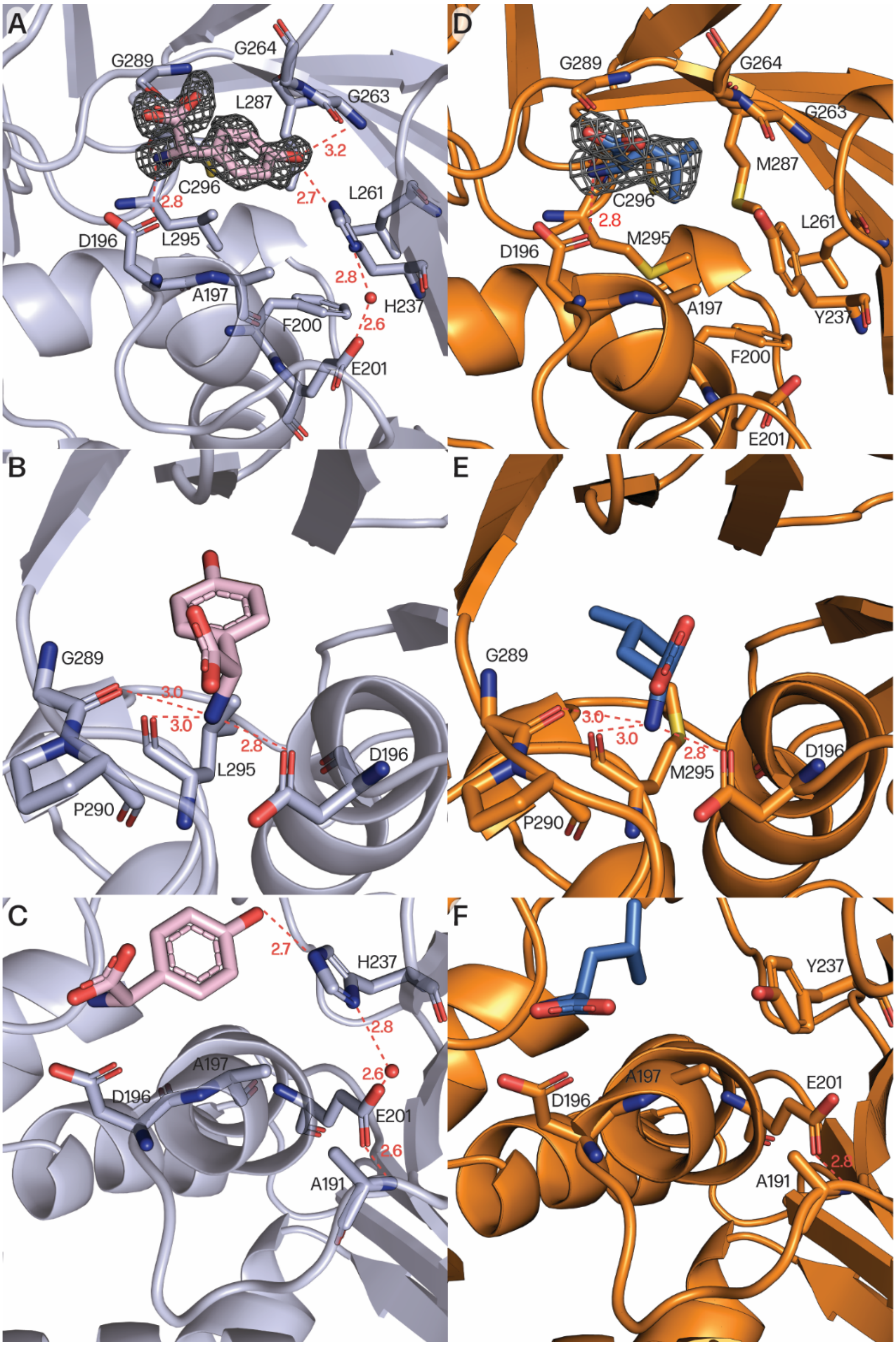

Figure S49. Substrate bound pocket of the A1$_{core-tei}$ and A1$_{core-ANC3}$. **A)** Close-up of the catalytic site of the ligand-bound A1$_{core-tei}$ structure in monomer A. Feature-enhanced electron density map[16] shown in dark grey of L-Hpg with L-Hpg omitted from the map calculation. The map, which is shown at a contour level of 1.5σ shows density consistent with the substrate occupying the binding pocket. The benzene ring of L-Hpg is stabilised by hydrophobic interactions by being sandwiched in between the sidechain of L295 and the main chain of G264. Finally, the 4-hydroxyl group of L-Hpg hydrogen bonds to H237 and to the amino group

of G263. **B)** Close-up of the catalytic site of the ligand-bound $A1_{core\text{-}tei}$ rotated 90° counterclockwise. The α-amino group of L-Hpg is coordinated by the formation of three hydrogen bonds, one to the side group of D196 and two hydrogen bonds to the carbonyl of the main chain carbonyl of L295 and G289. **C)** The H237 imidazole ring is oriented through a water-mediated interaction between E201 and the amino group next to the γ carbon of the imidazole ring. This Glu is widely conserved among NRPS A-domains with the purpose of hydrogen bonding and stabilising the loop immediately prior to the key α-amino coordinating acidic residue. **D)** View of $A1_{core\text{-}ANC3}$ substrate binding pocket with L-Leu bound. 2Fo–Fc feature-enhanced electron density map of ligand represented in dark grey and contoured at 1.5σ. **E)** View rotated 90° counter-clockwise. The α-amino group of L-Leu is coordinated by the formation of three hydrogen bonds, one to the side group of D196 and two hydrogen bonds to the carbonyl of the main chain carbonyl of L295 and G289. **F)** Upon mutation H237Y no coordinated water is observed between Y237 and E201.

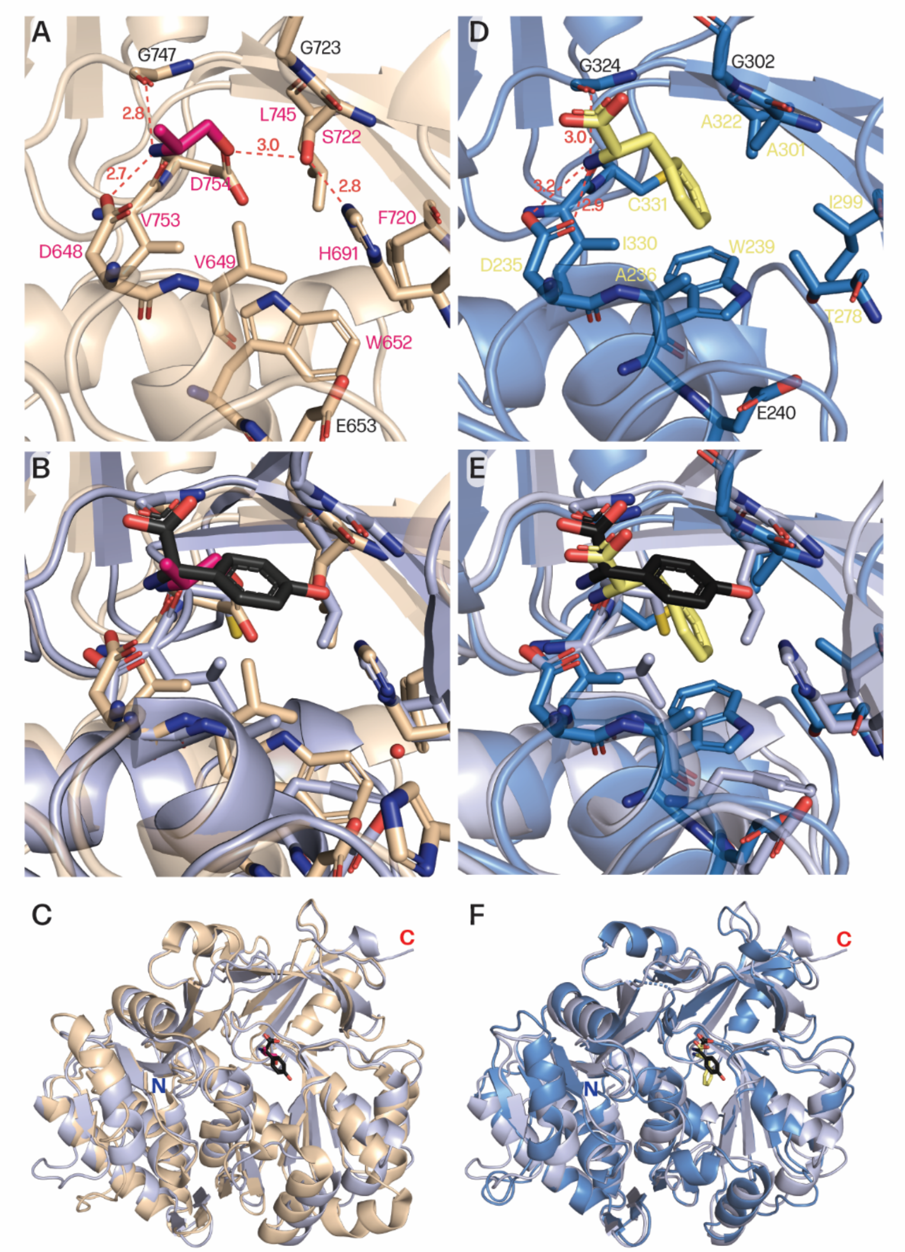

235	236	239	278	299	301	322	330	331	Position PDB:1AMU
D	A	W	T	I	A	A	I	C	Phe GrsA
648	649	652	691	720	722	745	753	754	Position PDB:5T3D
D	V	W	H	F	S	L	V	D	Ser EntF
196	199	200	237	261	263	287	295	296	Position
D	A	F	H	L	G	L	L	C	Hpg Tcp9 PDB:

Figure S50. Comparison of A1$_{core\text{-}tei}$ with EntF and PheA. **A)** Substrate binding pocket of EntF (PDB:5T3D). **B)** Overlay of the substrate-binding pockets of A1$_{core\text{-}tei}$ domain and EntF. **C)** Tcp9 A1$_{core\text{-}tei}$ and A$_{core}$ part of EntF superimposed. **D)** Substrate binding pocket of PheA (PDB:1AMU). **E)** Overlay of the substrate-binding pockets of A1$_{core\text{-}tei}$ and PheA. **F)** A1$_{core\text{-}tei}$ and A$_{core}$ part of PheA superimposed.

Supplementary Note 1

Supporting Data deposited on Zenodo (10.5281/zenodo.8410710)

Reconstructed sequence Paleomycin (node N1)
sequence_reconstructed_ancestral_GPA.fasta

Reconstructed sequences at nodes N4, N7 and N16
N4.fasta
N7.fasta
N16.fasta

Alignments used for paleomycin reconstruction
Mod_1-2_MAFFT_codon_alignment_stop_codons_removed_gaps_trimmed.fasta
Mod_3_mafft_codon_gaps_trimmed.fasta
Mod_4-6_MAFFT_codon_gaps_trimmed.fasta
Mod_7_MAFFT_codon_gaps_trimmed.fasta

Script for codon alignment of nucleotide sequences
Pal2nal.pl

Alignment and midpoint rooted guide tree (concatenated NRPS tree) used for ancestral state and ancestral sequence reconstruction (Used in Figure 4 and SI figures S2-6, S8, S10-11, S15, S17-18, S20-24, S26, S29)
Guide_tree_with_bootstraps.newick
Alignment_midpoint_rooted_guide_tree.fasta

Concatenated NRPS tree with outgroup rooting (Figure 3, Figure S1)
NRPS_tree_outgroup_bootstraps.newick
Rooted_guide_tree.newick

Species tree glycopeptide producers (Figure 3)
autoMLST_tree_GPA_producers.tree

Tanglegram species tree vs. NRPS tree (Figure 3)
Tanglegram_with_names.bmp

Sliding window analysis (Figure S30)
Sliding_window_analysis_Mod_1-3_300_100.xlsx

Posterior probabilities for the sequence reconstruction
probabilities_Mod_1-2.xlsx
probabilities_Mod_3.xlsx
probabilities_Mod_4-6.xlsx
probabilities_Mod_7.xlsx

Figures for ancestral sequence reconstruction and phylogeny of the biosynthesis enzymes:
Bht gene cassette (Figure S7)
BpsD_Bht_cassette.newick
Bhp_Bht_cassette.newick
OxyD_Bht_cassette.newick

P450 moonoxygenases (Figure S9)
P450_monooxygenases_OxyABCE.newick

Halogenases (Figure S12)
    Halogenases.newick

Glycosyltransferases (Figures S13 – 14)
    Gylcosyltransferases.newick
    GT_GPA_vs_CAZy_G1_MAFFT FastTree Tree.newick

Acyltransferases (Figure S16)
    Acyltransferases_1.newick
    Acyltransferases_2.newick

Methyltransferases (Figure S19 & S25)
    orthogroup_sequences_mtf_alignment_MAFFT.fasta.treefile
    RAxML_bestTree.Final_MT_dataset_RaxML.newick

Sulfotransferases (Figure S27)
    Sulfotransferases.newick

Vancosamine biosynthesis (Figure S28)
    EvaA_vancosamine.newick
    EvaB_vancosamine.newick
    EvaD_vancosamine.newick
    EvaE_vancosamine.newick

A- and C-domain phylogeny (Figure S43)
    GPA_A-domains.newick
    GPA_C-domains.newick

Full phylogeny of NRPSs producing a GPA scaffold and ancestral sequence reconstruction of A- domains (Figure S45)
    ASRguideTree.txt
    Bootstrapped_ASR_AACODONTREEalltrees.tree
    Max_probabilities_of_marginal_reconstruction_with_indels.txt
    probabilities_of_the_marginal_reconstruction_for_indels.txt
    probabilities_of_the_marginal_reconstruction_without_indels.txt
    sequences_of_the_marginal_reconstruction_including_indels.fas
    sequences_of_the_marginal_reconstruction_without_reconstruction_of_indels.fas
    Tree_in_Ancestor_format.txt
    Tree.txt

Supplementary References

1 MacNeil, D. J. *et al.* Analysis of Streptomyces avermitilis genes required for avermectin biosynthesis utilizing a novel integration vector. *Gene* **111**, 61-68, (1992).

2 Bennett, P. M., Grinsted, J. & Richmond, M. H. Transposition of TnA does not generate deletions. *Molecular and General Genetics MGG* **154**, 205-211, (1977).

3 Myronovskyi, M., Welle, E., Fedorenko, V. & Luzhetskyy, A. β-Glucuronidase as a Sensitive and Versatile Reporter in Actinomycetes. *Applied and Environmental Microbiology* **77**, 5370-5383, (2011).

4 Bibb, M. J., Janssen, G. R. & Ward, J. M. Cloning and analysis of the promoter region of the erythromycin resistance gene (ermE) of Streptomyces erythraeus. *Gene* **38**, 215-226, (1985).

5 Matsushima, P. & Baltz, R. H. A gene cloning system for 'Streptomyces toyocaensis'. *Microbiology* **142**, 261-267, (1996).

6 Kunzelmann, S. & Webb, M. R. A Biosensor for Fluorescent Determination of ADP with High Time Resolution. *Journal of Biological Chemistry* **284**, 33130-33138, (2009).

7 Assenberg, R. *et al.* Expression, purification and crystallization of a lyssavirus matrix (M) protein. *Acta Crystallographica Section F* **64**, 258-262, (2008).

8 Kaniusaite, M. *et al.* A proof-reading mechanism for non-proteinogenic amino acid incorporation into glycopeptide antibiotics. *Chemical Science* **10**, 9466-9482, (2019).

9 Chen, V. B. *et al.* MolProbity : all-atom structure validation for macromolecular crystallography. *Acta Crystallographica Section D* **66**, 12--21, (2010).

10 Huson, D. H. & Scornavacca, C. Dendroscope 3: an interactive tool for rooted phylogenetic trees and networks. *Systematic biology* **61**, 1061-1067, (2012).

11 Vaughan, T. G. IcyTree: rapid browser-based visualization for phylogenetic trees and networks. *Bioinformatics* **33**, 2392-2394, (2017).

12 Nguyen, L.-T., Schmidt, H. A., von Haeseler, A. & Minh, B. Q. IQ-TREE: a fast and effective stochastic algorithm for estimating maximum-likelihood phylogenies. *Molecular biology and evolution* **32**, 268-274, (2014).

13 Chernomor, O., von Haeseler, A. & Minh, B. Q. Terrace aware data structure for phylogenomic inference from supermatrices. *Systematic biology* **65**, 997-1008, (2016).

14 Kalyaanamoorthy, S., Minh, B. Q., Wong, T. K., von Haeseler, A. & Jermiin, L. S. ModelFinder: fast model selection for accurate phylogenetic estimates. *Nature methods* **14**, 587, (2017).

15 Soltis, P. S. & Soltis, D. E. Applying the bootstrap in phylogeny reconstruction. *Statistical Science*, 256-267, (2003).

16 Afonine, P. *et al.* FEM: feature-enhanced map. *Acta Crystallogr D Biol Crystallogr.* **71**, 646-666, (2015).
